# Patterns of intravenous fluid resuscitation use in adult intensive care patients between 2007 and 2014: An international cross-sectional study

**DOI:** 10.1371/journal.pone.0176292

**Published:** 2017-05-12

**Authors:** Naomi E. Hammond, Colman Taylor, Simon Finfer, Flavia R. Machado, YouZhong An, Laurent Billot, Frank Bloos, Fernando Bozza, Alexandre Biasi Cavalcanti, Maryam Correa, Bin Du, Peter B. Hjortrup, Yang Li, Lauralyn McIntryre, Manoj Saxena, Frédérique Schortgen, Nicola R. Watts, John Myburgh, for the Fluid-TRIPS and Fluidos Investigators, The George Institute for Global Health, The ANZICS Clinical Trials Group, BRICNet, and the REVA research Network

**Affiliations:** 1 Critical Care and Trauma Division, The George Institute for Global Health, Sydney, Australia; 2 Malcolm Fisher Department of Intensive Care Medicine, Royal North Shore Hospital, Sydney, Australia; 3 Sydney Medical School, University of Sydney, Sydney, Australia; 4 St George Clinical School, Faculty of Medicine, University of New South Wales, Sydney, Australia; 5 Anesthesiology, Pain and Intensive Care Department, Federal University of Sao Paulo, Sao Paulo, Brazil; 6 Department of Critical Care Medicine, Peking University People’s Hospital, Beijing, China; 7 Statistics Division, The George Institute for Global Health, Sydney, Australia; 8 Department of Anesthesiology and Intensive Care Medicine, Jena University Hospital, Jena, Germany; 9 D'Or Institute for Research and Education, Rio de Janeiro, Brazil; 10 Research Institute, Hospital do Coração, São Paulo, Brazil; 11 Medical Intensive Care Unit, Peking Union Medical College Hospital, Beijing, China; 12 Department of Intensive Care, Copenhagen University Hospital, Rigshospitalet, Denmark; 13 Department of Medicine (Critical Care), The Ottawa Hospital Research Institute, Ottawa, Canada; 14 Department of Intensive Care Medicine, St. George Hospital, Sydney, Australia; 15 Assistance Publique-Hôpitaux de Paris, Réanimation Médicale Groupe Hospitalier Henri Mondor, Créteil, France; University of Colorado Denver, UNITED STATES

## Abstract

**Background:**

In 2007, the Saline versus Albumin Fluid Evaluation—Translation of Research Into Practice Study (SAFE-TRIPS) reported that 0.9% sodium chloride (saline) and hydroxyethyl starch (HES) were the most commonly used resuscitation fluids in intensive care unit (ICU) patients. Evidence has emerged since 2007 that these fluids are associated with adverse patient-centred outcomes. Based on the published evidence since 2007, we sought to determine the current type of fluid resuscitation used in clinical practice and the predictors of fluid choice and determine whether these have changed between 2007 and 2014.

**Methods:**

In 2014, an international, cross-sectional study was conducted (Fluid-TRIPS) to document current patterns of intravenous resuscitation fluid use and determine factors associated with fluid choice. We examined univariate and multivariate associations between patients and prescriber characteristics, geographical region and fluid type. Additionally, we report secular trends of resuscitation fluid use in a cohort of ICUs that participated in both the 2007 and 2014 studies. Regression analysis were conducted to determine changes in the administration of crystalloid or colloid between 2007 and 2014.

**Findings:**

In 2014, a total of 426 ICUs in 27 countries participated. Over the 24 hour study day, 1456/6707 (21.7%) patients received resuscitation fluid during 2716 resuscitation episodes. Crystalloids were administered to 1227/1456 (84.3%) patients during 2208/2716 (81.3%) episodes and colloids to 394/1456 (27.1%) patients during 581/2716 (21.4%) episodes. In multivariate analyses, practice significantly varied between geographical regions. Additionally, patients with a traumatic brain injury were less likely to receive colloid when compared to patients with no trauma (adjusted OR 0.24; 95% CI 0.1 to 0.62; p = 0.003). Patients in the ICU for one or more days where more likely to receive colloid compared to patients in the ICU on their admission date (adjusted OR 1.75; 95% CI 1.27 to 2.41; p = <0.001).

For secular trends in fluid resuscitation, 84 ICUs in 17 countries contributed data. In 2007, 527/1663 (31.7%) patients received fluid resuscitation during 1167 episodes compared to 491/1763 (27.9%) patients during 960 episodes in 2014. The use of crystalloids increased from 498/1167 (42.7%) in 2007 to 694/960 (72.3%) in 2014 (odds ratio (OR) 3.75, 95% confidence interval (CI) 2.95 to 4.77; p = <0.001), primarily due to a significant increase in the use of buffered salt solutions. The use of colloids decreased from 724/1167 (62.0%) in 2007 to 297/960 (30.9%) in 2014 (OR 0.29, 95% CI 0.19 to 0.43; p = <0.001), primarily due to a decrease in the use of HES, but an overall increase in the use of albumin.

**Conclusions:**

Clinical practices of intravenous fluid resuscitation have changed between 2007 and 2014. Geographical location remains a strong predictor of the type of fluid administered for fluid resuscitation. Overall, there is a preferential use of crystalloids, specifically buffered salt solutions, over colloids. There is now an imperative to conduct a trial determining the safety and efficacy of these fluids on patient-centred outcomes.

**Trial registration:**

**Clinicaltrials.gov**: Fluid-Translation of research into practice study (Fluid-TRIPS) NCT02002013

## Introduction

Fluid resuscitation is a common intervention in the management of patients treated in the intensive care unit (ICU) where over one third of these patients receive intravenous fluid for haemodynamic resuscitation on any given day.[1] Over the last two decades there has been an evolving body of research directed at determining the safety and efficacy of resuscitation fluids. [2–10]

In 2007, our group conducted an international, cross-sectional study of 391 ICUs from 25 countries that reported that 0.9% sodium chloride (saline) and hydroxyethyl starch solutions (HES) were the most commonly used intravenous crystalloid and colloid solutions respectively. [1] Since 2007, a number of randomised trials[4,6–10] and observational studies have reported associations between the administration of specific intravenous resuscitation fluids and adverse patient-centred outcomes.[11–15]

Our objective was to describe current practices about the choice and use of fluid resuscitation by ICU clinicians; to examine factors associated with fluid choice and to compare secular trends in fluid resuscitation use between 2007 and 2014. Our hypothesis was that practice had changed as a result of recent clinical trial publications.

## Methods

We conducted a prospective, international, cross-sectional observational study in a convenience sample of ICUs in 2014. Sites were recruited via the collaborative network developed to conduct a cross-sectional study in 2007 –the Saline vs. Albumin Fluid Evaluation—Translation of Research into Practice Study (SAFE-TRIPS).[1] In addition, we directly contacted leaders of established international critical care networks and leading individual intensive care clinician-researchers to encourage associated ICUs to participate in the study. Ten potential study days between April 2014 and December 2014 were designated to facilitate logistics for individual sites to participate in one elected study day. The study day was defined as a 24 hour period according to the participating site’s daily ICU chart.

**Fig 1:**
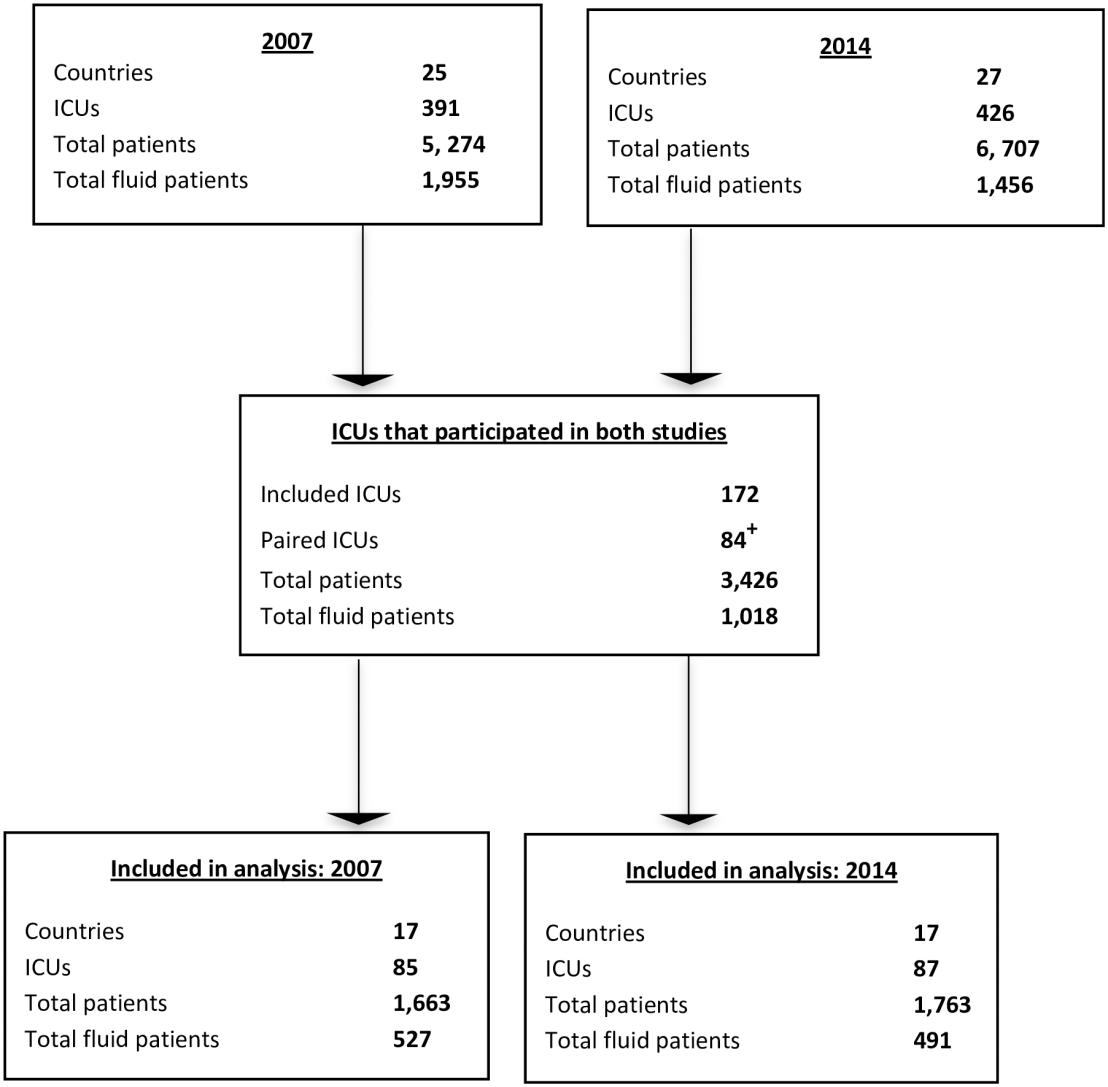
Flow diagram of included ICUs and patients in 2007 and 2014. + Some ICUs that contributed data to both studies may have been defined as a single ICU in one study but as two ICUs in another study therefore the ‘paired’ ICU number not half of the total ICU number. These ICUs were combined into one ICU (as appropriate) to enable comparison of fluid use over time.

For the comparison of secular trends in fluid resuscitation use between 2007 and 2014, ICUs that participated in both the 2007 SAFE-TRIPS study and this study were included (Fig 1).

The study protocol was first approved by the New South Wales Ethics Review Committee (RPAH Zone), Australia (Approval number X14-0061 and LNR/14/RPAH/72). For all other sites, Human Research Ethics Committee approval was obtained with either a waiver or written informed consent for data collection as per local requirements.

### Participants and data collection

We collected the number of patients being treated in the participating ICUs on the study day. Of these, those who received one or more fluid resuscitation episodes any time during the 24 hour study period were included. Patients less than 16 years of age were excluded.

Fluid resuscitation episodes were defined as an hour during which a patient received a specifically prescribed intravenous fluid bolus of any crystalloid or colloid solution; a continuous infusion of 5ml/kg/hr or greater of crystalloid and/or any dose of colloid by continuous infusion. This definition of continuous infusion of fluid was obtained by investigator consensus before the 2007 study and used in 2014 to draw consistent comparisons.

Using a standard case report form, data were collected on all patients who received fluid resuscitation present in the participating ICU for all or part of the 24-hour study day. Data was entered into an electronic data capture system (REDCap—Vanderbilt University, Tennessee, USA) [16] hosted at the George Institute for Global Health, Sydney, Australia, apart from Brazilian sites where the data capture system was hosted at Institute D’Or de Ensino e Pesquisa, Rio de Janerio, Brazil. Data were checked using pre-determined range limits and queries were resolved with the individual sites.

Patient data collected included demographics, admission source and diagnosis, severity of illness score (such as Acute Physiology And Chronic Health Evaluation (APACHE) II [17] or Simplified Acute Physiology Score (SAPS) II[18]), and number of days in the ICU (where the first day of ICU admission was designated as day 0).

For each episode of fluid resuscitation, the type of fluid infused; the indication(s) for the fluid defined as impaired perfusion or low cardiac output, ongoing bleeding, other non-haemorrhagic fluid losses, unit protocol, abnormal vital signs; the prescriber characteristics defined as resident, registrar, ICU specialist or nurse; the cardiovascular and respiratory component of the Sequential Organ Failure Assessment (SOFA) score;[19] physiological variables including heart rate, mean arterial pressure, central venous pressure; laboratory values including creatinine, bilirubin, lactate, albumin concentration; cumulative urine output and total fluid output in the previous complete hour; the use of mechanical ventilation and renal replacement therapy were recorded. The type of resuscitation fluid was classified as crystalloids (saline, buffered salt solutions or other crystalloids) or colloids (albumin, HES, gelatin, dextran solutions) (S1 File,S1 and S2 Tables).

### Statistical analyses

All analyses were carried out using R statistical software package (R version 3.1.0 (2014-04-10).[20] As more than one type of fluid could have been administered during a resuscitation episode, proportions could add to more than 100%.

Comparison of patient and prescriber characteristics for administration of crystalloids or colloids were tested using a t-test or Wilcoxon rank-sum test for continuous data or Pearson’s chi-squared for categorical data as appropriate. Differences in proportions of crystalloids and colloids used in fluid resuscitation episodes between geographical regions were tested using generalised estimating equations (GEEs), accounting for clustering at the patient level.

Multivariate analyses using GEEs accounting for clustering at the patient level, were conducted to determine associations between patient demographics, clinical characteristics and the type of fluid administered. Initially, each factor of interest was examined separately to determine if there was an association with the administered fluid. Variables meeting a pre-determined level of statistical significance (p <0·1) with the administration of crystalloid or colloid were included in the final model. Associations were considered statistically significant if p <0·01. Results of the multivariate analysis are presented as adjusted odds ratios (OR) and 95% confidence intervals (CI). Details regarding categorical data and handling of missing data are provided in S1 File.

Determination of the secular trends of types of fluid resuscitation use between 2007 and 2014 was completed by comparing patterns of fluid use in sites that participated in both the 2007 and 2014 studies (Fig 1). For differences over time in proportions of fluid indication and by fluid prescriber, GEEs were used to account for repeated episodes. Differences in fluid proportions over time were also analysed using GEEs accounting for clustering at the patient level and presented as unadjusted OR and 95% CI. A p value of <0·05 was considered to be statistically significant. Multivariate analysis, using GEEs were conducted to determine the change in the administration of crystalloid or colloid between the 2007 and the 2014 sample with methods as described above. Further details on the secular trend multivariate analysis methods are provided in the S1 File.

We examined secular trends in crystalloid and colloid use in six predefined subgroups: geographic region; an admission medical or surgical diagnosis; in patients with and without a diagnosis of sepsis in the 24 hours prior to the study day; in patients with and without an admission diagnosis of trauma (with or without traumatic brain injury (TBI)); in patients with high versus low severity of illness 24 hours prior to fluid administration; the number of days in the ICU at the survey day. For each subgroup we assessed heterogeneity of effect between time points by adding an interaction term between the two time periods and the subgroup variable of interest. P-values for heterogeneity of <0·05 was considered to be statistically significant. Further details on subgroup definitions are provided in S1 File.

One pre-specified sensitivity analysis of the initial fluid resuscitation episode only was conducted for both the overall 2014 data and the secular trend data. GEEs were used accounting for clustering at a hospital level to determine the effect of patient clustering by episode and that the results generated were consistent with the main analysis.

## Results

A total of 426 ICUs from 27 countries participated in the 2014 study. During the 24-hour study period, 1456/6707 (21.7%) patients received resuscitation fluid over 2716 fluid resuscitation episodes (Table 1). Characteristics of these patients according to crystalloid or colloid administration are presented in S3 Table. Of these, 446/1456 (30.7%) and 292/1456 (20.1%) patients received resuscitation fluid on day 0 or day 1 of their ICU admission respectively (S1 Fig). The indication for the fluid episodes and the fluid prescriber are presented in S4 Table.

**Table 1:** Countries, intensive care units, patients, and fluid resuscitation episodes.

Country	ICUs (N)	Total Patients (N)	Fluid Patients (N)	Fluid Patient (%)	Fluid Episodes (N)
**Argentina**	2	40	10	25.00%	11
**Australia**	25	500	125	25.00%	256
**Belgium**	2	57	13	22.81%	19
**Brazil**	217	3214	519	16.15%	880
**Canada**	11	268	69	25.75%	148
**China**	33	608	157	25.82%	238
**Denmark**	14	157	27	17.20%	63
**France**	23	329	78	23.40%	138
**Germany**	19	486	181	37.24%	396
**Greece**	1	7	4	57.14%	13
**India**	4	59	19	32.20%	31
**Italy**	8	49	20	40.82%	35
**Monaco**	1	8	0	NA	NA
**Netherlands**	1	10	6	60.00%	6
**New Zealand**	7	113	30	26.55%	56
**Norway**	6	40	12	30.00%	22
**Republic of Korea**	1	59	10	16.95%	23
**Saudi Arabia**	2	79	24	30.38%	56
**Singapore**	9	92	21	22.83%	45
**Slovakia Region**	1	6	2	33.33%	4
**South Africa**	2	28	13	46.43%	21
**Sweden**	6	60	23	38.33%	40
UK*	13	159	60	37.74%	159
**USA**	17	245	26	10.61%	47
**Vietnam**	1	34	7	20.59%	9
**All**	**426**	**6,707**	**1,456**	**21.69%**	**2,716**

*Includes ICUs from England, Scotland and Northern Ireland (no participating ICUs from Wales)

**Fig 2:**
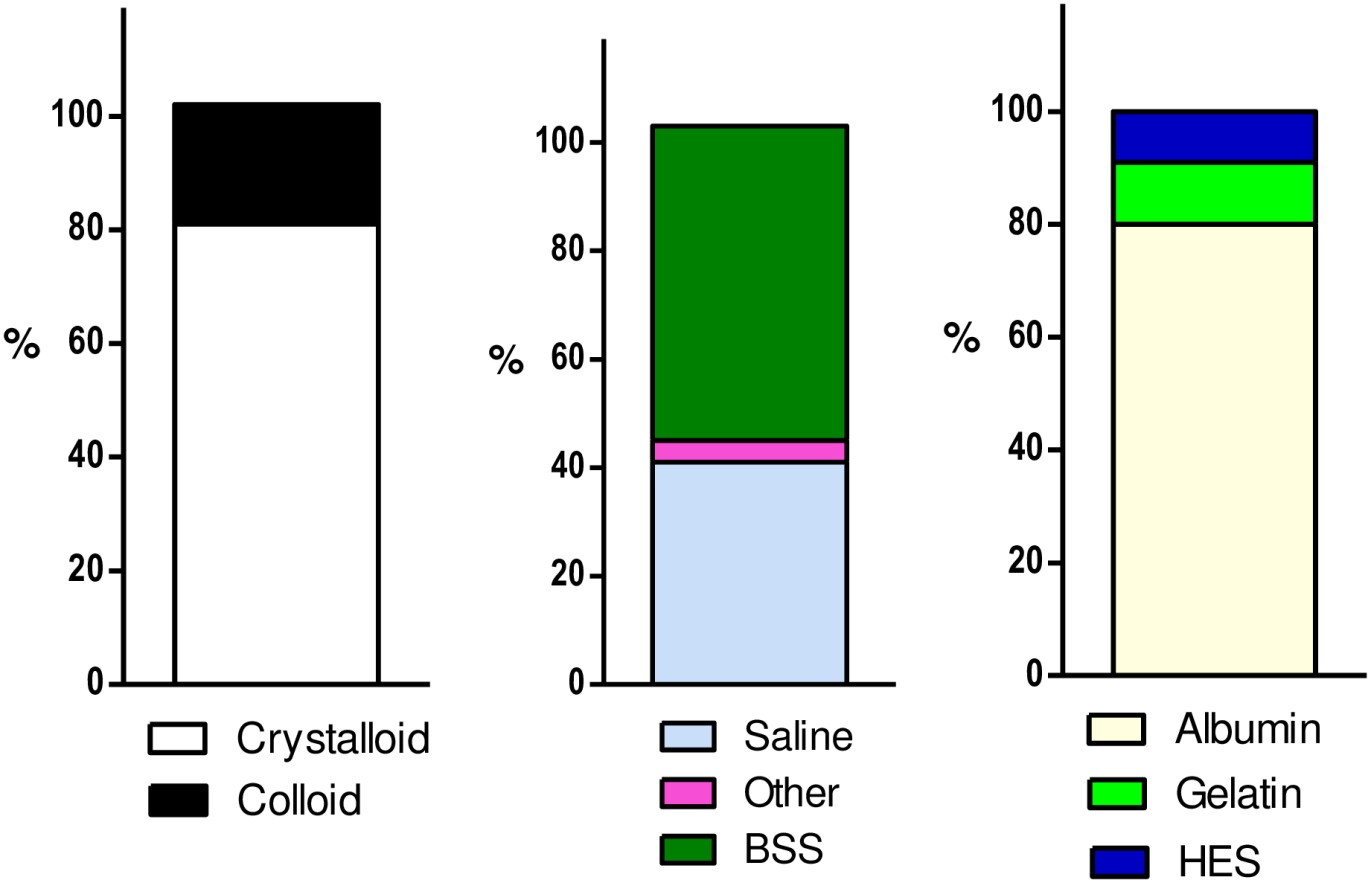
Proportion of all fluid resuscitation episodes of crystalloid and colloid in 2014 in 426 ICUs. Proportions may not add to 100% as patients can be administered more than one type of fluid during resuscitation episodes. Denominator for crystalloid and colloid panel is all fluid resuscitation episodes (n = 2716); Denominator for crystalloid panel is for all crystalloid episodes (n = 2208); Denominator for colloid panel is for all colloid episodes (n = 581). BSS = Buffered Salt Solutions. HES = Hydroxyethyl Starch. Other = other crystalloids.

Crystalloids were administered to 1227/1456 (84.3%) patients during 2208/2716 (81.3%) episodes and colloids to 394/1456 (27.1%) patients during 581/2716 (21.4%) episodes (Fig 2). There was significant variation between geographical region with the proportion of crystalloid use ranging from 50.8% to 95.8% and the proportion of colloid use ranging from 6.3% to 60.5% (S2 Fig).

For all crystalloid resuscitation episodes, buffered salt solutions were administered in 1280/2208 (58.0%) episodes and saline in 897/2208 (40.6%) episodes (Fig 2; S3 Fig).

For all colloid resuscitation episodes, albumin was administered in 463/581 (79.7%) episodes; gelatin in 64/581 (11.0%) episodes and HES in 51/581 (8.8%) episodes (Fig 2; S4 Fig).

Patient characteristics, physiological variables and prescriber factors associated with the administration of crystalloid and colloid are shown by patient and by episodes of fluid resuscitation in S5 Table.

After adjusting for factors that were found to be associated with the administration of crystalloid or colloid, the type of fluid prescribed differed significantly between geographical locations (S6 Table).

Three clinical factors were found to be significantly associated with the type of fluid administration. Trauma patients with TBI were less likely to receive colloid (OR 0.24; 95%CI 0.1 to 0.62; p = 0.003) than patients without trauma and TBI. Fewer patients received crystalloids after their admission day to the ICU (OR 0.46; 95% CI 0.32 to 0.66; p = <0.001), with more patients receiving colloids (OR 1.75; 95% CI 1.27 to 2.41; p = <0.001), compared to day zero. Crystalloids were administered in fewer episodes according to the ICU protocol (OR 0.4; 95%CI 0.2 to 0.8; p = <0.001), compared to the indication of ‘impaired perfusion or low cardiac output’ (S6 Table).

For the secular trends of fluid resuscitation, a total of 84 ICUs from 17 countries participated in both the 2007 and 2014 studies (Fig 1, Table 2).The number of patients receiving fluid resuscitation in 2007 was 527/1663 (31.7%) during 1167 fluid resuscitation episodes compared to 491/1763 (27.9%) patients during 960 fluid resuscitation episodes in 2014 (Table 2).

**Table 2:** Countries, intensive care units, and patients included in comparison between 2007 and 2014.

Country	ICUs (N)	Paired ICUs (N)	2007 Total Patients (N)	2014 Total Patients (N)	Total Patients Overall (N)	2007 Fluid Patients (N)	2014 Fluid Patients (N)	Fluid Patient Overall (N)	Fluid Patients Overall (%)	2007 Fluid Episodes (N)	2014 Fluid Episodes (N)	Fluid Episodes Overall (N)
**Australia**	26	13	264	313	577	74	73	147	25.5%	173	143	316
**Brazil**	6	3	78	89	167	9	11	20	12.0%	10	24	34
**Canada**	15	7	222	195	417	76	56	132	31.7%	172	113	285
**China**	36	18	404	407	811	151	116	267	32.9%	253	180	433
**Denmark**	18	9	71	94	165	30	19	49	29.7%	56	50	106
**France**	19	9	171	134	305	34	36	70	23.0%	64	67	131
**Germany**	12	6	181	215	396	69	68	137	34.6%	249	160	409
**India**	2	1	31	16	47	8	10	18	38.3%	16	17	33
**Italy**	4	2	19	19	38	6	9	15	39.5%	12	16	28
**New Zealand**	10	5	66	95	161	26	26	52	32.3%	80	48	128
**Norway**	3	1	8	13	21	4	3	7	33.3%	4	8	12
**Saudi Arabia**	2	1	25	63	88	6	16	22	25.0%	19	31	50
**Singapore**	5	2	29	28	57	4	8	12	21.1%	4	17	21
**Sweden**	4	2	13	19	32	3	9	12	37.5%	5	14	19
UK*	10	5	81	63	144	27	31	58	40.3%	50	72	122
**All**	**172**	**84**	**1,663**	**1,763**	**3,426**	**527**	**491**	**1,018**	**29.7%**	**1,167**	**960**	**2,127**

*Includes ICUs from England, Scotland and Northern Ireland

Patient characteristics in the 2007 and 2014 studies were similar apart from a significant difference in ICU stay on the survey day: median 3 days (interquartile range [IQR] 0 to 10) versus 1 day (0 to 7) respectively; p = <0.001. (S7 Table)). Comparisons of indication for the fluid episodes and the fluid prescriber in the 2007 and 2014 studies are presented in S8 Table.

**Fig 3:**
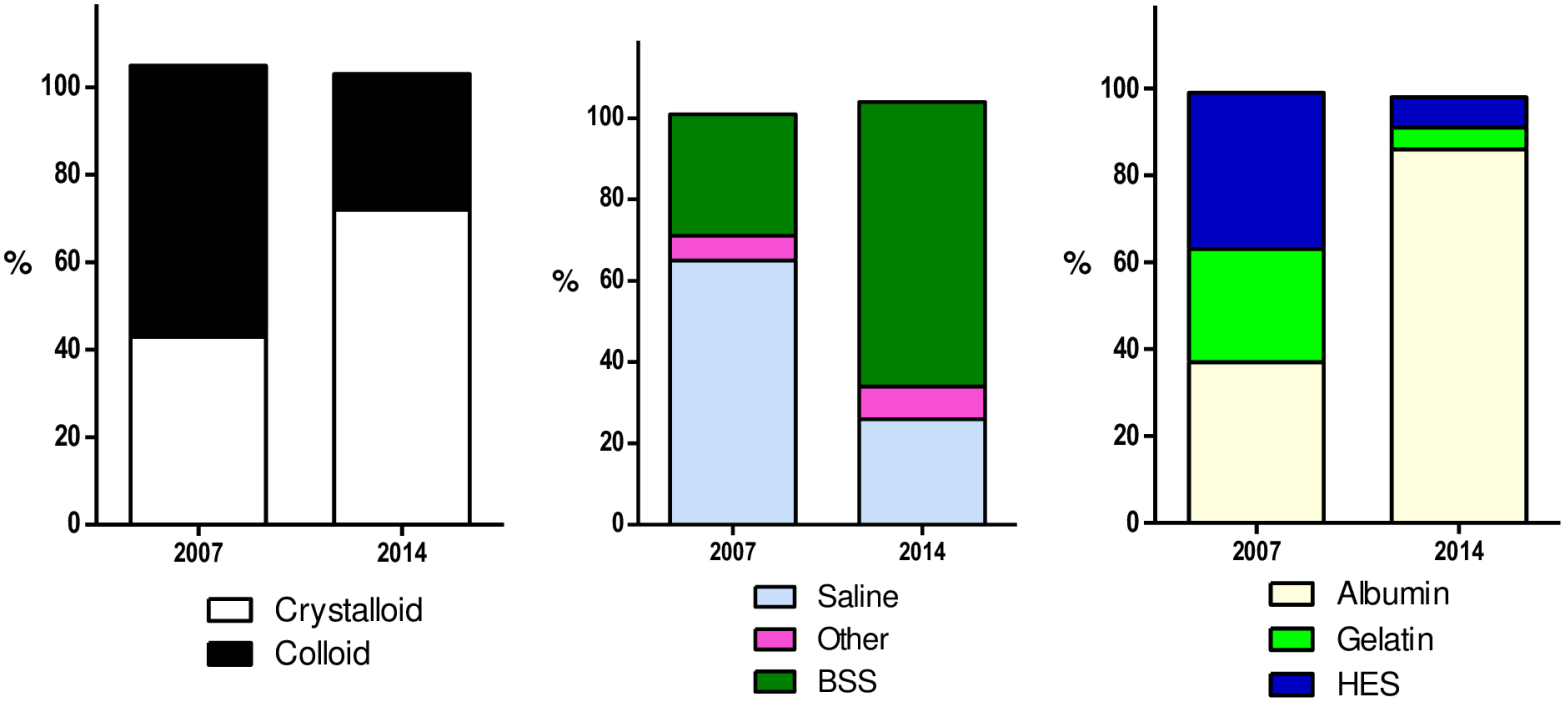
Proportion of all fluid resuscitation episodes given in 2007 and 2014 in 84 ICUs. Denominator for crystalloid and colloid panel is all fluid resuscitation episodes (n = 1167 in 2007 and n = 960 in 2014); Denominator for crystalloid panel is for crystalloid episodes only (n = 498 in 2007 and n = 694 in 2014); Denominator for colloid panel is for all colloid episodes (n = 724 in 2007 and n = 297 in 2014). Proportions may not add to 100% as patients can be administered more than one type of fluid during resuscitation episodes. BSS = Buffered Salt Solutions. HES = Hydroxyethyl Starch. Other = other crystalloids.

**Fig 4:**
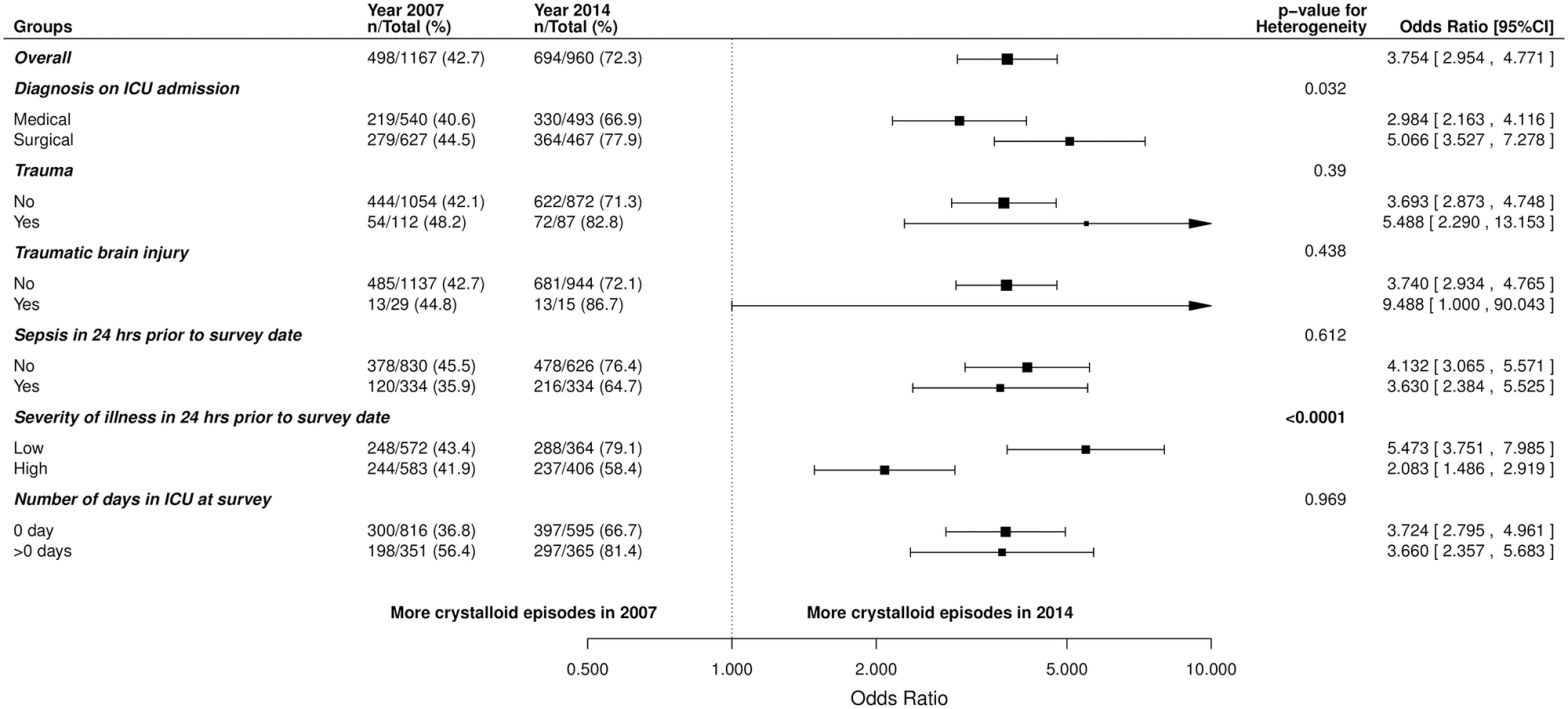
Forest plots of change in use of crystalloid fluid resuscitation episodes between 2007 and 2014; overall and by pre-defined subgroup. Unadjusted odds ratios and 95% Confidence Intervals (CI) presented.

The proportion of patients who received crystalloids increased significantly between 2007 and 2014; 240/527 (45.5%) in 2007 versus 365/491 (74.3%) in 2014 (OR 2.98, 95% CI 2.02 to 4.40; p = <0.001). crystalloids were administered in significantly more episodes; 498/1167 (42.7%) in 2007 versus 694/960 (72.3%) in 2014 (OR 3.75, 95% CI 2.95 to 4.77; p = <0.001) (Fig 3). Variations in the patterns of fluid resuscitation administration in the participating ICUs in the different geographical regions are presented between 2007 and 2014 (Fig 4; S5–S7 Figs). In the multivariate analysis, the trends were consistent after adjusting for significant univariates (OR 3.46, 95% CI 2.59 to 4.64; p = <0.001) (Table 3).

**Table 3:** Results of multivariate and sensitivity analysis for receiving crystalloid and colloid fluid resuscitation episodes in 2014 compared to 2007.

**Multivariate analysis (all fluid resuscitation episodes)**^a^				
**Characteristic**	**OR (95%CI) for receiving crystalloid**	**P value**	**OR (95%CI) for receiving colloid**	**P value**
**Study**				
** 2007**	1.00		1.00	
** 2014**	3.46 (2.59 to 4.64)	<0.001	0.28 (0.21 to 0.38)	<0.001
**Sensitivity analysis (first fluid resuscitation episode only)**^b^				
**Characteristic**	**OR(95%CI) for receiving crystalloid**	**P value**	**OR(95%CI) for receiving colloid**	**P value**
**Study**				
**2007**	1.00		1.00	
**2014**	2.22 (1.33 to 3.70)	0.002	0.43 (0.26 to 0.72)	0.001

^a^ Results are generated from GEE model with patient ID as a cluster. Analysis include 1,979 episodes and 947 study participants as data were lost due to missing values which could not be included in the multivariate analysis. This number represents a loss of 7.0% of episodes and 7.0% of study participants.

^b^ Results are generated from GEE model with site/ICU ID as a cluster. Analysis include 848 first fluid episodes from 848 study participants as data were lost due to missing values which could not be included in the multivariate analysis. This number represents a loss of 16.7% of first fluid episodes/study participants.

Among all crystalloid episodes, the use of buffered salt solutions increased significantly between 2007 and 2014: 150/498 (30.1%) in 2007 versus 484/694 (69.7%) in 2014 (OR 3.26, 95% CI 2.35 to 4.52; p = <0.001); the use of saline decreased significantly: 326/498 (65.5%) in 2007 versus 183/694 (26.4%) in 2014 (OR 0.33, 95% CI 0.24 to 0.46; p = <0.001) (Fig 3).

**Fig 5:**
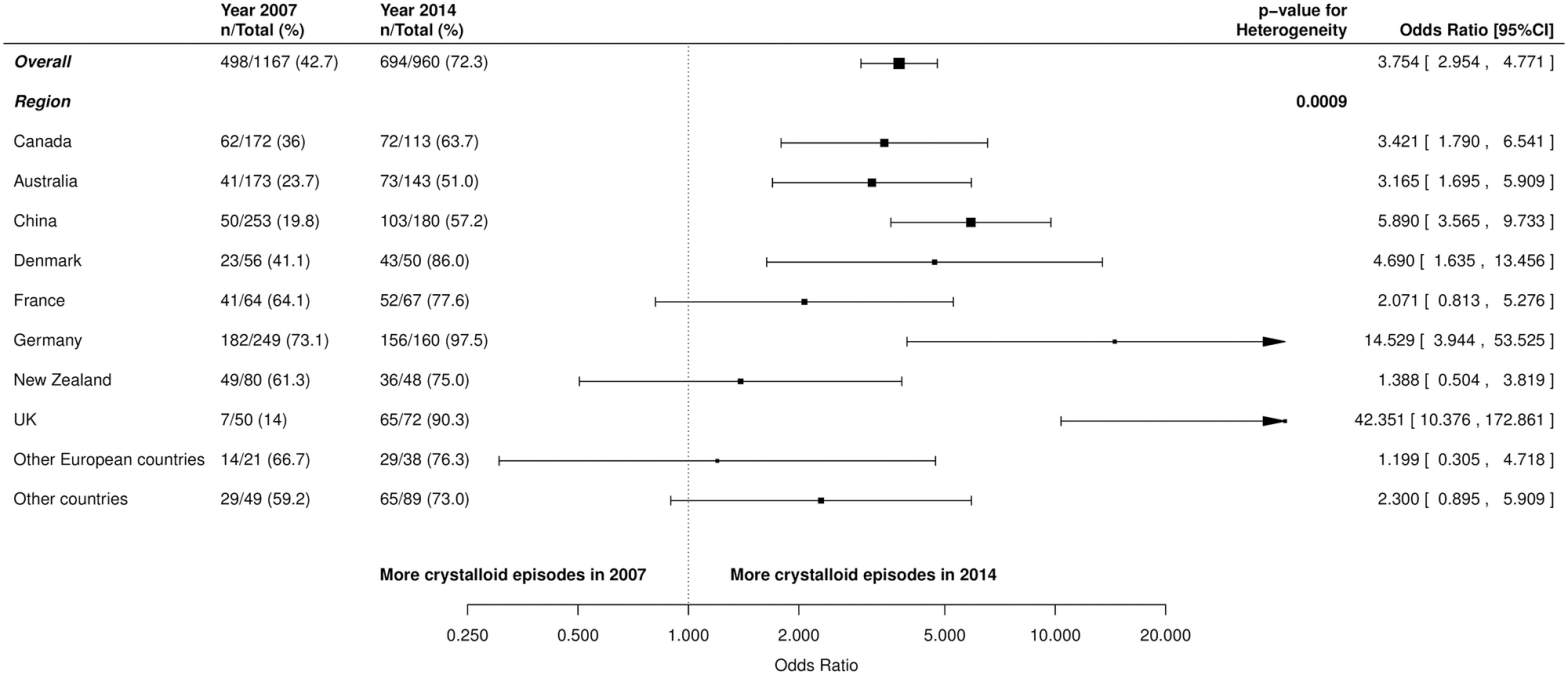
Forest plots of crystalloid fluid resuscitation episodes between 2007 and 2014; overall and by region subgroup. Unadjusted odds ratios and 95% Confidence Intervals (CI) presented.

More patients received crystalloids in 2014 compared to 2007 in all subgroups, with significant heterogeneity between medical versus surgical admissions (OR 2.98, 95% CI 2.16 to 4.12 versus OR 5.07, 95% CI 3.53 to 7.28 respectively; p = 0.03), and low versus high severity of illness (OR 5.47, 95% CI 3.75 to 7.99 versus OR 2.08, 95% CI 1.49 to 2.91 respectively; p = <0.001) (Fig 4). For geographical region, the change in crystalloid administration varied significantly in the participating sites (p<0.001) (Fig 5).

The proportion of patients who received colloid decreased significantly between 2007 and 2014: 390/527 (74.0%) in 2007 versus 205/491 (41.8%) in 2014 (OR 0.29, 95% CI 0.19 to 0.43; p = <0.001); these were administered in significantly fewer episodes: 724/1167 (62.0%) in 2007 versus 297/960 (30.9%) in 2014 (OR 0.27, 95% CI 0.22 to 0.35, p = <0.001) (Fig 3). In the multivariate analysis, the trends were consistent after adjusting for significant univariates (OR 0.28, 95% CI 0.21 to 0.38; p = <0.001) (Table 3).

Fewer patients received colloids in all subgroups in 2014 compared to 2007, with significant heterogeneity between medical versus surgical admissions (OR 0.34, 95% CI 0.25 to 0.47 versus OR 0.21, 95% CI 0.15 to 0.30 respectively; p = 0.04), and low versus high severity of illness (OR 0.20, 95% CI 0.14 to 0.29 versus OR 0.47, 95% CI 0.33 to 0.65 respectively; p = <0.001) (S8 Fig). For geographical region there was significant variation in colloid administration in participating sites (p = <0.001) (S9 Fig).

The use of albumin increased significantly between 2007 and 2014: 272/724 (37.6%) in 2007 versus 257/297 (86.5%) in 2014 (OR 8.86, 95% CI 5.87 to 13.37; p = <0.001). The use of HES decreased significantly between 2007 and 2014: 256/724 (35.4%) in 2007 versus 22/297 (7.4%) in 2014 (OR 0·16, 95% CI 0.10 to 0.25; p = <0.001); as did the use of gelatin 185/724 (25.6%) in 2007 versus 16/297) (5.4%) in 2014 (OR 0.24, 95% CI 0.13 to 0.44; p = <0.001), and the use of dextran 23/724 (3.18%) in 2007 versus 2/297 (0.67%) in 2014 (Fig 3).

The sensitivity analysis of the initial fluid resuscitation episode for the 2014 study (S9 Table) and the secular trends in crystalloid and colloid use between 2007 and 2014 were consistent with the main analyses: OR 2.22, 95% CI 1.33 to 3.70; p = 0.002 for crystalloid use in 2014 versus OR 0.43, 95% CI 0.26 to 0.72; p = 0.001 for colloid use in 2014 (Table 3).

## Discussion

In this international, cross-sectional study, one fifth of patients in the ICU received intravenous resuscitation fluid and if the study day coincided with the patient’s admission date, close to one third of patients received resuscitation fluid. Overall, crystalloids were administered to more patients and during more episodes than colloids, driven by a preferential and increased use of buffered salt solutions. The geographical region in which the patient was being treated was a significant determinant of fluid choice.

In 2013, an international observational study recording only the first fluid challenge given in consecutive ICU adult patients reported that crystalloids were administered in more than 70% of all fluid episodes, with buffered salt solutions administered in more than half of these episodes. [21] An observational study conducted by our group reported similar secular trends in fluid resuscitation over a seven year period (2007–2013) in Australian and New Zealand ICUs.[22] The majority of evidence to support the change in clinical practice to the use of buffered salt solutions is from observational and registry based studies.[11,13,14,23] Recent evidence from a cluster cross-over randomised trial that compared a proprietary buffered salt solution (Plasma-Lyte 148^®^, Baxter Healthcare, Australia) to saline did not find a difference between fluid groups on the primary outcome of risk of acute kidney injury or the secondary outcomes of renal replacement therapy and in-hospital mortality.[24] Of note the majority of patients included in the SPLIT trial were elective surgical admissions to the ICU and the volumes of fluid administered were small, raising the possibility of a type II error. As SPLIT was published after our survey, we were unable to examine the impact of the results from this study on clinical practice.

When comparing fluid resuscitation practices in the same international cohort of ICUs over a 7 year period, there was a significant increase in the use of crystalloids and a significant decrease in the use of colloid in 2014 when compared to 2007. These changes were influenced by an increased use of buffered salt solutions and decreased use of semi-synthetic colloids in 2014. Although there was variability between geographical regions, in patients with medical and surgical diagnoses and patients with increased severity of illness, the patterns of increased crystalloid and decreased colloid administration from 2007 to 2014 were observed across all patient subgroups. Of note, patients with a higher severity of illness received significantly less crystalloid than patients with low severity of illness between 2007 and 2014.The drivers for the observed change in clinical practice are likely to be multifactorial, but potentially associated with evidence from pivotal randomised-controlled trials that reported adverse patient outcomes associated with the administration of HES solutions and the subsequent changes to licencing by Medical Regulatory Authorities; product availability in the different regions and recommendations in updated clinical practice guidelines. [25–28]

When colloids were administered, albumin was the predominant colloid used in both the overall 2014 data and in the secular trend data. This observation may relate to reduced use of HES after 2007 and emerging evidence of potential benefit of albumin in patients with severe sepsis. [5] [10]

Geographical region was a significant determinant of the type of fluid administered, which is consistent with findings from the 2007 study. [1] Although we have demonstrated this effect is independent of patient and prescriber characteristics, other potential confounders such as fluid availability, cost, and hospital policy are likely to have an influence.

Our study was conducted during a time where a number of high profile fluid resuscitation trials were published, changes in the availability of intravenous fluids and changes to clinical practice recommendations occurred. We used standard case report forms and definitions that are consistent with other observational studies of fluid resuscitation conducted by our group. [1,22] We collected detailed information on clinical factors that may potentially influence the choice of fluid for resuscitation at the time fluid episodes were administered thereby enabling analyses to account for patient and prescriber characteristics. We mitigated selection bias by using data from the same ICUs at the two time points and confounding bias by adjusting for potential and statistically determined confounders according to a pre-specified statistical analysis plan. The sensitivity analysis confirmed that the first fluid episode was representative of subsequent fluid episodes received over the study day and that multiple episodes over the study day did not alter the observed trends. We did not account for the availability of fluids in participating hospitals and regions that may have contributed to the observed changes in fluid use over time. We recognise the use of convenience sampling limits the external validity of our findings outside these units and regions and that as some countries were under and over-represented in our 2014 sample, of particular note is the number of contributing ICUs from Brazil was just over half of all participating ICUs. The definition of fluid resuscitation used in these two studies are at variance to some current fluid resuscitation guidelines where larger volumes are recommended,[29] but the definitions used were designed by consensus to draw comparisons of secular changes. Interpretation of fluid usage in specific patient populations, such as traumatic brain injury, require caution due to relatively small patient numbers.

Future research should be directed to determine and understand actual and potential drivers behind clinician selection of resuscitation fluid. Based on our study findings and despite established changes in clinical practice, definitive randomised controlled trials comparing saline to buffered salt solutions are warranted to inform clinicians, regulators and policy makers on the relative efficacy and safety of these fluids.[30]

## Conclusion

Fluid resuscitation practices have changed between 2007 and 2014. Crystalloid use, predominantly buffered salt solutions, has increased significantly while colloid use has decreased, predominantly due to decreased use of HES. Geographical location remains a strong predictor of the type of fluid administered for fluid resuscitation.

## Supporting information

S1 File Supplementary methods. (PDF)

S1 Table Crystalloid and colloid fluid types collected. (PDF)

S2 Table Fluid indication hierarchy used in analysis. (PDF)

S3 Table Characteristics of 1,456 fluid patients in relation to administration of crystalloid or colloid. (PDF)

S4 Table Indication for fluid and fluid prescriber for 2716 fluid resuscitation episodes. (PDF)

S5 Table Univariate analysis of factors associated with administration of crystalloid or colloid for 2716 fluid resuscitation episodes in 1456 patients. (PDF)

S6 Table Multivariate analysis of factors associated with the use of crystalloid and colloid for fluid resuscitation episodes in 2014. (PDF)

S7 Table Comparison of patient characteristics of 527 fluid patients in 2007 and 491 fluid patients in 2014. (PDF)

S8 Table Comparison of indication for fluid and fluid prescriber between 1167 fluid resuscitation episodes in 2007 and 960 fluid resuscitation episodes in 2014. (PDF)

S9 Table Sensitivity analysis—Multivariate analysis of factors associated with the use of crystalloid or colloid for the first fluid resuscitation episode in 2014. (PDF)

S1 Fig Proportion of patients receiving fluid resuscitation according to the number of days in the ICU at the study day. (TIF)

S2 Fig Percentage of all fluid resuscitation episodes given in 2014 as crystalloid or colloid by geographical region (n = 2716). (TIF)

S3 Fig Type of crystalloid used in 2014 as a percentage of all crystalloid episodes by geographical region (n = 2208). (TIF)

S4 Fig Type of colloid used in 2014 as a percentage of all colloid episodes by geographical region (n = 581). (TIF)

S5 Fig Proportion of fluid resuscitation episodes given as crystalloid and colloid in 2007 (n = 1167) and 2014 (n = 960) by geographical region. (TIF)

S6 Fig Percentage of crystalloid fluid resuscitation episodes given in 2007 (n = 498) and 2014 (n = 694) by geographical region. (TIF)

S7 Fig Percentage of colloid fluid resuscitation episodes given in 2007 (n = 724) and 2014 (n = 297) by geographical region. (TIF)

S8 Fig Forest plot of change in use of colloid fluid resuscitation episodes between 2007 and 2014; overall and by pre-defined subgroup. (TIF)

S9 Fig Forest plot of colloid fluid resuscitation episodes between 2007 and 2014; overall and by region subgroups. (TIF)
