# Supplementary material for: Patterns of intravenous fluid resuscitation use in adult intensive care patients between 2007 and 2014: An international cross-sectional study
Source: PLoS One. 2017 May 12;12(5):e0176292. doi: 10.1371/journal.pone.0176292 (PMC5428917; doi:10.1371/journal.pone.0176292)
Supplement: S1 File — (PDF) [file pone.0176292.s001.pdf]

S1 File: Supplementary Methods

Definition of Doctor Specialty level

A specialist doctor (consultant or attending) was a doctor who has completed advanced training in intensive care and/or applicable board or college registration. A registrar (including fellow or senior trainee) was a doctor who is undertaking advanced training in intensive care and/or applicable board or college registration. A resident (including a house medical officer or Junior Trainee) was a doctor who has more than one year of post graduate experience but has not yet entered advanced training in their specialty. The 'other' group was defined as an Intern/House Officer which is a junior doctor who has less than one year of post-graduate experience.

Fluid Indication hierarchy

In cases where more than one indication was selected for fluid resuscitation, they were classified according to a pre-determined hierarchy which is presented in table S

Subgroup definitions

Geographical region was determined by participating countries. The United Kingdom consists of contributing ICUs from England, Northern Ireland and Scotland. Countries with fluid resuscitation episodes of less than 50 were combined into two categories, 'other European countries' (Belgium, Greece, Italy, Netherlands, Norway, Slovakia Region, Sweden), and 'other countries' (Argentina, India, Republic of South Korea, Singapore, South Africa, USA, Vietnam).

For the secular trend data the countries with fluid resuscitation episodes of less than 50 were 'other European countries' (Italy, Norway, Sweden), and 'other countries' (Brazil, India, Saudi Arabia, Singapore, South Africa).

Sepsis was defined as; a defined focus of infection and two or more systemic inflammatory response syndrome (SIRS) criteria. Trauma was defined by a primary ICU admission diagnosis of trauma (an injury to the body produced by mechanical forces or a primary admission diagnosis of burns) or primary admission diagnosis of trauma plus TBI (confirmed by abnormality on cranial CT consistent with acute traumatic brain injury) or trauma without TBI (e.g. TBI not confirmed on CT, or intracranial haemorrhage on cranial CT). For pragmatic reasons, contributing sites could use the severity of illness score that was most often used in their ICU. We used the median of the severity of illness scores to dichotomise between high and low severity of illness subgroup.

Handling of missing data and univariate analysis

Variables that had more than 20% missing were analysed with a 'missing' category. Univariate analyses were conducted as described in the main paper with variables meeting a pre-determined level of statistical significance ($p < 0.1$) with the administration of crystalloid or colloid then included in the final multivariate model.

The univariate analysis used in the secular trend data was from the overall 2014 sample (Fluid-TRIPS). In cases of high collinearity between two variables, i.e. Pearson correlation coefficient >0.8 between continuous variables and Cramér's V statistics >0.8 between categorical variable, the

variable with a smaller univariate generalized estimating equation P value was included in the multivariate analyses. A sensitivity univariate analysis was also conducted in the same method as described for the main analysis to generate variables to be included in the sensitivity multivariate analysis (*Table S4*). This sensitivity univariate analysis is not presented.

Selection of reference group for categorical data

For categorical data with no natural order, the reference group was selected based on the category with the greatest number of observations, except for country, where France was selected because the pattern of fluid administered most closely resembled that of all the contributing ICUs combined. For the secular trend data Canada was selected because the pattern of fluid administered most closely resembled that of all the contributing ICUs.
