# Supplementary material for: Patterns of intravenous fluid resuscitation use in adult intensive care patients between 2007 and 2014: An international cross-sectional study
Source: PLoS One. 2017 May 12;12(5):e0176292. doi: 10.1371/journal.pone.0176292 (PMC5428917; doi:10.1371/journal.pone.0176292)
Supplement: S1 Table — (PDF) [file pone.0176292.s002.pdf]

S1 Table. Crystalloid and colloid fluid types collected

Crystalloid groups	Fluid type
**0.9% sodium chloride**	‘normal saline’ **/** ‘saline’
**Buffered (balanced) salt solutions (BSS)**	Hartmann’s, Plasmalyte R®, Plasmalyte 148®, Plasmalyte A®, lactated Ringer’s, Ringer’s acetate, Ringer’s simple®
**Other Crystalloids**	Hypertonic saline (>0.9% e.g. 3%, 7%, 7.5%, 20%); Glucose or glucose/saline (e.g. 5% glucose, 5% glucose in water, 4%N/5, 3.75%N/4, 2.5%N/2, Plasmalyte M®); Hypertonic glucose (e.g. >5% eg 10%, 20%, 50% dextrose); Sodium Bicarbonate
**Colloid groups**	**Fluid type**
**Albumin**	4% (or 5%), 20% (or 25%)
**Hydroxyethyl starch**	6% Hydroxyethyl starch (130/0.4x) in saline, 6% Hydroxyethyl starch (130/0.4x) in buffered salt solutions; Other hydroxyethyl starch preparations
**Gelatins**	Gelofusin®, Haemaccel®
**Dextran**	Dextran 40 or 70 (incl. macrodex®, rheomacrodex®
