# Supplementary material for: Patterns of intravenous fluid resuscitation use in adult intensive care patients between 2007 and 2014: An international cross-sectional study
Source: PLoS One. 2017 May 12;12(5):e0176292. doi: 10.1371/journal.pone.0176292 (PMC5428917; doi:10.1371/journal.pone.0176292)
Supplement: S2 Table — (PDF) [file pone.0176292.s003.pdf]

S2 Table. Fluid indication hierarchy used in analysis

**1. Impaired perfusion OR low cardiac output**	**YES to any of:**<br>Low urine output (3.08)<br>Low measured cardiac output (3.09-3.11)<br>Low SvO2/ScvO2 (3.13)<br>Clinical signs of poor peripheral perfusion (3.12)<br>Increasing or persisting acidosis or lactate (3.17)<br>Positive Straight Leg Raise test (3.18)<br>Abnormal indices of Pulse Pressure Variation (3.19)
**2. Ongoing bleeding**	**NO to all of:**<br>Low urine output (3.08)<br>Low measured cardiac output (3.09-3.11)<br>Low ScO2/ScvO2 (3.13)<br>Clinical signs of poor peripheral perfusion (3.12)<br>Increasing or persisting acidosis or lactate (3.17)<br>Positive Straight Leg Raise test (3.18)<br>Abnormal indices of Pulse Pressure Variation (3.19)<br>**YES to:**<br>Ongoing bleeding (3.14)<br>Other = Anemia<br>Other = Coagulopathy
**3. Other (non-hemorrhagic) fluid losses**	**NO to all of:**<br>Low urine output (3.08)<br>Low measured cardiac output (3.09-3.11)<br>Low ScO2/ScvO2 (3.13)<br>Clinical signs of poor peripheral perfusion (3.12)<br>Increasing or persisting acidosis or lactate (3.17)<br>Positive Straight Leg Raise test (3.18)<br>Abnormal indices of Pulse Pressure Variation (3.19)<br>Ongoing bleeding (3.14)<br>Other = Anemia<br>Other = Coagulopathy<br>**YES to:**<br>Other ongoing fluid loss (3.15)
**4. Unit protocol**	**NO to all of:**<br>Low urine output (3.08)<br>Low measured cardiac output (3.09-3.11)<br>Low ScO2/ScvO2 (3.13)<br>Clinical signs of poor peripheral perfusion (3.12)<br>Increasing or persisting acidosis or lactate (3.17)<br>Positive Straight Leg Raise test (3.18)<br>Abnormal indices of Pulse Pressure Variation (3.19)<br>Ongoing bleeding (3.14)<br>Other ongoing fluid loss (3.15)<br>Other = Anemia<br>Other = Coagulopathy<br>**YES to any of:**<br>Unit protocol or standing orders (3.16)
**5. Abnormal vital signs only**	**NO to all of:**<br>Low urine output (3.08)<br>Low measured cardiac output (3.09-3.11)<br>Low ScO2/ScvO2 (3.13)<br>Ongoing bleeding (3.14)<br>Other ongoing fluid loss (3.15)<br>Unit protocol or standing orders (3.16)<br>Clinical signs of poor peripheral perfusion (3.12)<br>Increasing or persisting acidosis or lactate (3.17)<br>Positive Straight Leg Raise test (3.18)<br>Abnormal indices of Pulse Pressure Variation (3.19)<br>**YES to any of:**<br>Hypotension (3.03)<br>Increasing inotrope or vasopressor requirements (3.04)<br>Low CVP (3.05)<br>Low PCWP (3.06)<br>Tachycardia (3.07)
**6. Other**	**NO to:**<br>3.03 – 3.19<br>**YES to:**<br>Other (3.20) unless it falls into one of the other specified categories

Table S2: Number next to the fluid indication relates to the case report form data number.
