# Supplementary material for: Patterns of intravenous fluid resuscitation use in adult intensive care patients between 2007 and 2014: An international cross-sectional study
Source: PLoS One. 2017 May 12;12(5):e0176292. doi: 10.1371/journal.pone.0176292 (PMC5428917; doi:10.1371/journal.pone.0176292)
Supplement: S3 Table — (PDF) [file pone.0176292.s004.pdf]

S3 Table. Characteristics of 1,456 fluid patients in relation to administration of crystalloid or colloid

		Crystalloid			Colloid		
**Variable**	**N**	**Yes**	**No**	**P Value**	**Yes**	**No**	**P Value**
**Patient Characteristics**							
**Age, year, median(IQR)]**	1456	64(50,74)	65(55,76)	0.023	64(53,74)	64(50,75)	0.461
**Male, %(N)**	878	59.2(726)	66.4(152)	0.041	65(256)	58.6(622)	0.027
**Number of days in the ICU (N=1455)**							
**Number of days in ICU median(IQR)]**	1455	1(0,6)	3(1,10)	<0.001	2(0,7)	1(0,6)	0.091
**Number of days in ICU at survey date =0 day, %(N)**	446	33.5(411)	15.4(35)	<0.001	28.5(112)	31.5(334)	0.278
**Number of days in ICU at survey date >0 day, %(N)**	1009	66.5(816)	84.6(193)		71.5(281)	68.5(728)	
**Severity of illness scores in 24 hrs prior to survey date (N=1456)**							
**APACHE II [median(IQR)]**	628	18(12,25)	21(15,26)	0.003	18(12,24)	18(12,25)	0.638
**SAPS II [median(IQR)]**	233	44(31.2,57)	49(43.5,68.5)	0.177	48.5(34.8,69)	44(31,56.5)	0.143
**SAPS III [median(IQR)]**	233	52(37,66.5)	65(48.2,72)	0.244	59(48,71)	51(37,66)	0.085
**Severity of illness , Low(< median), %(N)**	556	39.4(484)	31.4(72)	0.006	37.1(146)	38.6(410)	0.011
**Severity of illness , High(>= median), %(N)**	593	39(478)	50.2(115)		46.2(182)	38.7(411)	
**Severity of illness , Missing, %(N)**	307	21.6(265)	18.3(42)		16.8(66)	22.7(241)	
**Trauma (N=1453)**							
**Trauma at hospital admission, Yes, %(N)**	142	10.6(130)	5.3(12)	0.013	6.6(26)	10.9(116)	0.014
**Traumatic brain injury, Yes, %(N)**	37	2.9(35)	0.9(2)	0.083	1(4)	3.1(33)	0.025
**Sepsis in 24 hrs prior to survey date (N=1453)**							
**Sepsis, Yes, %(N)**	550	36(441)	47.8(109)	<0.001	43(169)	35.9(381)	0.014
**APACHE II chronic health points (CHP) criteria (N=1447), %(N)**							
**CHP liver criteria, Yes**	56	3.3(40)	7.1(16)	0.007	6.2(24)	3.1(32)	0.007
**CHP renal criteria, Yes**	33	2.3(28)	2.2(5)	0.936	1.8(7)	2.5(26)	0.439
**CHP cardiac criteria, Yes**	88	5.8(70)	8(18)	0.202	8.2(32)	5.4(56)	0.045
**CHP respiratory criteria, Yes**	92	5.5(67)	11(25)	0.002	7.6(30)	5.9(62)	0.235
**CHP immunocompromised, Yes**	157	11(134)	10.2(23)	0.703	11.8(46)	10.6(111)	0.522
**Admission Source (N=1455), %(N)**							
**Operating room after elective surgery**	380	26.8(329)	22.3(51)	<0.001	27.4(108)	25.6(272)	0.003
**Emergency room**	330	24.1(295)	15.3(35)		16.2(64)	25.1(266)	
**Hospital floor**	252	16.7(205)	20.5(47)		19(75)	16.7(177)	
**Transferred from other ICU or hospital**	166	10.6(130)	15.7(36)		13.7(54)	10.6(112)	
**Operating room after emergency surgery**	204	14.4(176)	12.2(28)		12.7(50)	14.5(154)	
**Hospital floor after previous ICU stay**	123	7.4(91)	14(32)		10.9(43)	7.5(80)	

Summary statistics of continuous variables are presented as median (IQR) with p values of non-parametric test (i.e. Wilcoxon rank-sum test). Summary statistics of categorical variables are presented in proportions with p values of chi^2 test. Categorical variables with ≥ 20% missing values are analysed with missing as separate groups.
