# Supplementary material for: Patterns of intravenous fluid resuscitation use in adult intensive care patients between 2007 and 2014: An international cross-sectional study
Source: PLoS One. 2017 May 12;12(5):e0176292. doi: 10.1371/journal.pone.0176292 (PMC5428917; doi:10.1371/journal.pone.0176292)
Supplement: S4 Table — (PDF) [file pone.0176292.s005.pdf]

S4 Table. Indication for fluid and fluid prescriber for 2716 fluid resuscitation episodes

Variable	% (N)<br>(N = 2697)		Variable	% (N)<br>(N = 2716)
**Indication for fluid in each fluid resuscitation**			**Fluid prescriber**	
**Impaired perfusion OR low cardiac output**	61.4 (1655)		Specialist (consultant/attending)	48.6 (1321)
**Ongoing bleeding**	2.3 (63)		Registrar (mid-level)	29.4 (798)
**Other fluid losses**	4 (108)		Resident (junior)	18.3 (497)
**Unit protocol**	5 (134)		Nurse and other	3.7 (100)
**Abnormal vital signs**	25.7 (693)			
**Fluid indication, other**	1.6 (44)			
