# Supplementary material for: Patterns of intravenous fluid resuscitation use in adult intensive care patients between 2007 and 2014: An international cross-sectional study
Source: PLoS One. 2017 May 12;12(5):e0176292. doi: 10.1371/journal.pone.0176292 (PMC5428917; doi:10.1371/journal.pone.0176292)
Supplement: S5 Table — (PDF) [file pone.0176292.s006.pdf]

S5 Table. Univariate analysis of factors associated with administration of crystalloid or colloid for 2716 fluid resuscitation episodes in 1456 patients

Variable	OR[95%CI] for receiving crystalloid	p_value	OR[95%CI] for receiving colloid	p_value
**Age (per one year increase)**	0.99[0.98 to 1]	0.0070	1.01[1 to 1.01]	0.0710
**Gender**				
**Female**	1.00		1.00	
**Male**	1.34[1.04 to 1.73]	0.0247	0.73[0.58 to 0.93]	0.0103
**Number of days in ICU at survey date**	0.99[0.99 to 1]	0.0724	1.01[1 to 1.01]	0.1032
**Number of days in ICU at survey**				
**0 day**	1.00		1.00	
**>0 day**	0.56[0.42 to 0.73]	<0.0001	1.54[1.2 to 1.98]	0.0007
**Severity of illness in 24 hrs prior to survey date**				
**Low(< median)**	1.00	0.0068	1.00	0.0052
**High(>= median)**	0.67[0.51 to 0.88]		1.39[1.08 to 1.8]	
**Missing**	0.99[0.7 to 1.4]		0.88[0.63 to 1.23]	
**Trauma at hospital admission Yes/No**				
**Trauma at hospital admission, No**	1.00		1.00	
**Trauma at hospital admission, Yes**	1.85[1.14 to 3.01]	0.0125	0.55[0.35 to 0.85]	0.0079
**Traumatic brain injury Yes/No**				
**Traumatic brain injury, No**	1.00		1.00	
**Traumatic brain injury, Yes**	3.37[0.97 to 11.67]	0.0556	0.29[0.1 to 0.87]	0.0277
**Trauma category at hospital admission**				
**No trauma**	1.00	0.0362	1.00	0.0186
**Trauma without TBI**	1.58[0.94 to 2.67]		0.65[0.4 to 1.05]	
**Trauma with TBI**	3.47[1 to 12.02]		0.28[0.1 to 0.85]	
**Sepsis in 24 hrs prior to survey date**				
**Sepsis in 24 hrs prior to survey date, No**	1.00		1.00	
**Sepsis in 24 hrs prior to survey date, Yes**	0.66[0.52 to 0.85]	0.0010	1.47[1.16 to 1.85]	0.0014
**Chronic health points liver criteria**				
**No**	1.00		1.00	
**Yes**	0.44[0.26 to 0.74]	0.0023	2.04[1.21 to 3.42]	0.0073
**Chronic health points renal criteria**				
**No**	1.00		1.00	
**Yes**	1.06[0.46 to 2.47]	0.8901	0.77[0.33 to 1.8]	0.5514
**Chronic health points cardiac criteria**				
**No**	1.00		1.00	
**Yes**	0.58[0.37 to 0.9]	0.0163	1.63[1.06 to 2.53]	0.0274
**Chronic health points respiratory criteria**				
**No**	1.00		1.00	
**Yes**	0.61[0.39 to 0.96]	0.0314	1.36[0.87 to 2.14]	0.1814
**Chronic health points immunocompromised**				
**No**	1.00		1.00	
**Yes**	1.01[0.68 to 1.48]	0.9782	1.05[0.73 to 1.51]	0.7827

**Admission source**				
**Operating room after elective surgery**	1.00	0.0014	1.00	0.0033
**Emergency room**	0.55[0.37 to 0.83]		1.59[1.09 to 2.33]	
**Hospital floor**	0.46[0.29 to 0.72]		2.05[1.34 to 3.13]	
**Transferred from other ICU or hospital**	0.8[0.51 to 1.26]		1.25[0.82 to 1.9]	
**Operating room after emergency surgery**	0.75[0.51 to 1.1]		1.34[0.94 to 1.91]	
**Hospital floor after previous ICU stay**	0.45[0.27 to 0.73]		2.16[1.36 to 3.42]	
**Indication for fluid**				
**Impaired perfusion/low cardiac output**	1.00	<0.0001	1.00	<0.0001
**Ongoing bleeding**	0.67[0.31 to 1.46]		1.57[0.82 to 3]	
**Other fluid losses**	0.68[0.4 to 1.13]		1.66[1.04 to 2.65]	
**Unit protocol**	0.22[0.12 to 0.41]		4.11[2.3 to 7.33]	
**Abnormal vital signs**	0.74[0.58 to 0.95]		1.22[0.96 to 1.54]	
**Fluid indication, other**	0.23[0.09 to 0.56]		3.76[1.6 to 8.84]	
**Fluid prescriber**				
**Specialist**	1.00	0.0002	1.00	<0.0001
**Registrar**	0.58[0.45 to 0.74]		1.84[1.45 to 2.32]	
**Resident**	1.01[0.72 to 1.43]		1.04[0.76 to 1.42]	
**Nurse**	2.8[0.15 to 52.37]		0.36[0.03 to 4.42]	
**Fluid prescriber, other**	0.91[0.38 to 2.18]		1.22[0.57 to 2.62]	
**Cardiac failure**				
**No (cardiac SOFA<3)**	1.00		1.00	
**Yes (cardiac SOFA>=3)**	0.84[0.67 to 1.05]	0.1167	1.21[0.98 to 1.5]	0.0779
**Respiratory failure**				
**No (respiratory SOFA<3)**	1.00		1.00	
**Yes (respiratory SOFA>=3)**	0.79[0.61 to 1.03]	0.0832	1.43[1.12 to 1.83]	0.0043
**Renal replacement therapy**				
**No**	1.00		1.00	
**Yes**	0.54[0.38 to 0.76]	0.0005	1.92[1.37 to 2.67]	0.0001
**Mechanical ventilation**				
**No**	1.00		1.00	
**Yes**	0.61[0.49 to 0.77]	<0.0001	1.61[1.29 to 2.01]	<0.0001
**Low filling pressure**				
**No,**	1.00	<0.0001	1.00	<0.0001
**Yes**	1.8[0.86 to 3.74]		0.89[0.48 to 1.64]	
**Missing**	1.73[1.36 to 2.19]		0.55[0.44 to 0.69]	
**Metabolic acidosis**				
**No**	1.00	0.1767	1.00	0.3005
**Yes**	1.22[0.97 to 1.54]		0.85[0.68 to 1.05]	
**Missing**	1[0.8 to 1.26]		0.97[0.79 to 1.21]	
**Lactate(mmol/L)**				
**<2,**	1.00	0.1406	1.00	0.8615
**>=2**	1.22[0.97 to 1.54]		0.94[0.76 to 1.17]	
**missing**	0.98[0.79 to 1.22]		0.99[0.8 to 1.22]	

**Heart rate, b/min**	1[1 to 1.01]	0.1725	1[1 to 1]	0.9384
**Heart rate (per 10 b/min increase)**	1.03[0.99 to 1.08]	0.1657	1[0.96 to 1.04]	0.9740
**Mean arterial pressure**	1[0.99 to 1]	0.3293	1[1 to 1.01]	0.5181
**Mean arterial pressure (per 10 mmHg decrease)**	1.03[0.98 to 1.09]	0.1999	0.97[0.93 to 1.02]	0.2864
**Creatinine(umol/L)**				
**<170**	1.00	0.6113	1.00	0.7008
**>=170**	0.99[0.73 to 1.33]		1.04[0.78 to 1.39]	
**missing**	0.9[0.72 to 1.11]		1.1[0.89 to 1.36]	
**Bilirubin(umol/L)**				
**<20**	1.00	0.0038	1.00	0.0020
**>=20**	1.08[0.82 to 1.43]		1[0.76 to 1.3]	
**missing**	1.47[1.16 to 1.85]		0.69[0.54 to 0.87]	
**Albumin(g/L)**				
**<27**	1.00	<0.0001	1.00	<0.0001
**>=27**	0.76[0.56 to 1.03]		1.28[0.96 to 1.71]	
**missing**	1.59[1.18 to 2.15]		0.61[0.46 to 0.8]	
**Urine output(ml/kg/hr)**				
**<0.5**	1.00	0.1275	1.00	0.3264
**>=0.5**	0.95[0.74 to 1.23]		1.1[0.87 to 1.4]	
**missing**	1.22[0.91 to 1.66]		0.94[0.71 to 1.24]	
**Fluid output(ml/kg/hr)**				
**<1**	1.00	0.0032	1.00	0.0054
**>=1**	1.08[0.82 to 1.43]		1.02[0.78 to 1.32]	
**missing**	1.63[1.16 to 2.3]		0.7[0.51 to 0.97]	

Continuous variables with ≥ 20% missing values are dichotomised and analysed with missing as a separate groups. Categorical variables with ≥ 20% missing values are also analysed with missing as a separate groups. Results are generated from generalised estimating equation (GEE) models with patient ID assigned as clusters. Variables with p-values from GEE <0.1 were eligible to be included in multivariate analysis.
