# Supplementary material for: Patterns of intravenous fluid resuscitation use in adult intensive care patients between 2007 and 2014: An international cross-sectional study
Source: PLoS One. 2017 May 12;12(5):e0176292. doi: 10.1371/journal.pone.0176292 (PMC5428917; doi:10.1371/journal.pone.0176292)
Supplement: S6 Table — (PDF) [file pone.0176292.s007.pdf]

S6 Table. Multivariate analysis of factors associated with the use of crystalloid and colloid for fluid resuscitation episodes in 2014

Characteristic	OR (95%CI) for receiving crystalloid	p_value	OR (95%CI) for receiving colloid	p_value
**Study region**		<0.001		<0.001
**France**	1.00		1.00	
**Australia**	0.16 (0.07 to 0.34)		4.16 (2.05 to 8.42)	
**Brazil**	6.69 (3.19 to 14.07)		0.19 (0.1 to 0.36)	
**Canada**	0.41 (0.18 to 0.95)		1.75 (0.79 to 3.88)	
**China**	0.20 (0.09 to 0.41)		6.96 (3.49 to 13.88)	
**Denmark**	0.68 (0.25 to 1.88)		1.02 (0.37 to 2.8)	
**Germany**	3.36 (1.37 to 8.23)		0.33 (0.15 to 0.71)	
**New Zealand**	0.31 (0.11 to 0.9)		2.28 (0.84 to 6.18)	
**Saudi Arabia**	0.57 (0.19 to 1.68)		1.87 (0.68 to 5.12)	
**UK**	1.46 (0.49 to 4.38)		0.44 (0.15 to 1.28)	
**Other European countries**	0.28 (0.12 to 0.64)		2.63 (1.21 to 5.70	
**Other countries**	0.61 (0.28 to 1.37)		1.46 (0.7 to 3.06)	
**Age (per one year increase)**	0.99 (0.98 to 1)	0.026	1.01 (1 to 1.02)	0.156
**Gender**		0.108		0.063
**Female**	1.00		1.00	
**Male**	0.77 (0.56 to 1.06)		1.33 (0.98 to 1.79)	
**Number of days in ICU at survey date**		<0.001		<0.001
**Number of days in ICU at survey date =0 day**	1.00		1.00	
**Number of days in ICU at survey date >0 day**	0.46 (0.32 to 0.66)		1.75 (1.27 to 2.41)	
**Severity of illness in 24 hrs prior to survey date**				
**Low(< median)**	1.00	0.259	1.00	0.097
**High(>= median)**	0.93 (0.65 to 1.33)		1.05 (0.75 to 1.47)	
**Missing**	1.37 (0.84 to 2.24)		0.66 (0.42 to 1.03)	
**Trauma at hospital admission**				
**No Trauma**	1.00	0.086	1.00	0.006
**Trauma without TBI**	1.25 (0.68 to 2.28)		0.64 (0.35 to 1.15)	
**Trauma with TBI**	3.29 (1.11 to 9.74)		0.24 (0.1 to 0.62)	
**Sepsis in 24 hrs prior to survey date**				
**No**	1.00		1.00	
**Yes**	0.82 (0.59 to 1.14)	0.2412	1.12 (0.81 to 1.54)	0.489
**Chronic health point liver criteria**				
**No**	1.00		1.00	
**Yes**	0.40 (0.19 to 0.8)	0.010	1.99 (1.05 to 3.75)	0.035
**Chronic health point cardiac criteria**				
**No**	1.00		1.00	
**Yes**	0.75 (0.42 to 1.34)	0.335	1.26 (0.74 to 2.16)	0.392
**Chronic health point respiratory criteria**				
**No**	1.00		1.00	
**Yes**	1.07 (0.61 to 1.89)	0.815	0.74 (0.42 to 1.32)	0.307

**Admission source**				
**Operating room after elective surgery**	1.00	0.072	1.00	0.023
**Emergency room**	1.58 (0.93 to 2.67)		0.68 (0.42 to 1.08)	
**Hospital floor**	1.06 (0.64 to 1.76)		0.82 (0.51 to 1.32)	
**Transferred from other ICU or hospital**	0.83 (0.5 to 1.39)		1.17 (0.72 to 1.9)	
**Operating room after emergency surgery**	1.10 (0.65 to 1.84)		0.94 (0.57 to 1.52)	
**Hospital floor after previous ICU stay**	0.61 (0.32 to 1.16)		1.78 (1 to 3.15)	
**Indication for fluid**				
**Impaired perfusion or low cardiac output**	1.00	0.070	1.00	0.382
**Ongoing bleeding**	0.53 (0.18 to 1.55)		1.33 (0.49 to 3.6)	
**Other fluid losses**	1.11 (0.58 to 2.12)		0.91 (0.49 to 1.7)	
**Unit protocol**	0.40 (0.2 to 0.8)		1.97 (1 to 3.88)	
**Abnormal vital signs**	0.88 (0.64 to 1.22)		1.00 (0.74 to 1.34)	
**Indication for fluid, other**	0.36 (0.1 to 1.32)		1.88 (0.51 to 6.94)	
**Fluid prescriber**				
**Specialist**	1.00	0.485	1.00	0.393
**Registrar**	1.09 (0.76 to 1.57)		0.94 (0.67 to 1.32)	
**Resident**	1.25 (0.79 to 1.97)		0.83 (0.55 to 1.26)	
**Nurse**	4.38 (0.56 to 34.25)		0.23 (0.03 to 1.71)	
**Fluid prescriber, other**	0.90 (0.3 to 2.68)		1.37 (0.53 to 3.52)	
**Cardiovascular dysfunction**				
**No (SOFA<3)**	1.00		1.00	
**Yes (SOFA>=3)**	1.07 (0.78 to 1.46)	0.672	0.99 (0.74 to 1.33)	0.960
**Respiratory dysfunction**				
**No (SOFA<3)**	1.00		1.00	
**Yes (SOFA>=3)**	1.32 (0.96 to 1.81)	0.093	0.92 (0.68 to 1.24)	0.572
**Renal replacement therapy**				
**No**	1.00		1.00	
**Yes**	0.59 (0.37 to 0.93)	0.022	1.77 (1.13 to 2.77)	0.013
**Mechanical ventilation**				
**No**	1.00		1.00	
**Yes**	0.65 (0.47 to 0.91)	0.012	1.36 (0.98 to 1.87)	0.063
**Low filling pressure**				
**No**	1.00	0.173	1.00	0.260
**Yes**	2.50 (0.74 to 8.43)		0.97 (0.39 to 2.4)	
**missing**	1.24 (0.89 to 1.72)		0.77 (0.55 to 1.05)	
**Bilirubin**				
**<20 umol/L**	1.00	0.142	1.00	0.057
**>=20 umol/L**	0.69 (0.47 to 1)		1.56 (1.08 to 2.23)	
**missing**	0.89 (0.59 to 1.35)		1.12 (0.76 to 1.66)	
**Albumin**				
**<27 g/L**	1.00	0.997	1.00	0.783
**>=27 g/L**	1.01 (0.69 to 1.49)		0.89 (0.61 to 1.29)	
**missing**	1.00 (0.62 to 1.61)		0.87 (0.55 to 1.37)	

**Fluid output**				
**<1 ml/kg/hr**	1.00	0.973	1.00	0.737
**>=1 ml/kg/hr**	0.97 (0.67 to 1.4)		1.14 (0.81 to 1.61)	
**missing**	0.95 (0.61 to 1.48)		1.15 (0.76 to 1.74)	

Results are generated from a generalised estimating equation model with patient ID as a cluster. The P-values displayed in the table are type III P-values. Analysis include 2,463 episodes and 1,317 study participants as data were lost due to missing values which could not be included in the multivariate analysis. This number represents a loss of 9.3% of episodes and 9.5% of study participants.
