# Supplementary material for: Patterns of intravenous fluid resuscitation use in adult intensive care patients between 2007 and 2014: An international cross-sectional study
Source: PLoS One. 2017 May 12;12(5):e0176292. doi: 10.1371/journal.pone.0176292 (PMC5428917; doi:10.1371/journal.pone.0176292)
Supplement: S7 Table — (PDF) [file pone.0176292.s008.pdf]

S7 Table. Comparison of patient characteristics of 527 fluid patients in 2007 and 491 fluid patients in 2014

Variable	2007		2014		
	N	Summary statistics	N	Summary statistics	p value
Demographic characteristics					
Age, year [median(IQR)]	527	66.0(52,75)	491	63.0(53,74)	0.147
Male, %(N)	527	67.2(354)	491	62.5(307)	0.121
Number of days in ICU	527		491		
Number of days in ICU [median(IQR)]		3.0(0,10)		1.0(0,7)	<.001
Admission source (%,N)	526		491		
Operating room after elective surgery		27.6(145)		24.2(119)	0.154
Emergency room		16.2(85)		21.8(107)	
Hospital floor		21.7(114)		18.3(90)	
Transferred from other ICU or hospital		11.0(58)		13.2(65)	
Operating room after emergency surgery		14.8(78)		13.6(67)	
Hospital floor after previous ICU stay		8.7(46)		8.8(43)	
Surgical diagnosis on admission, Yes %(N)	527	47.6(251)	491	44.0(216)	0.245
Trauma	526		490		
Trauma at hospital admission, Yes %(N)		10.5(55)		9.2(45)	0.496
Traumatic brain injury, Yes %(N)		3.2(17)		2.0(10)	0.238
Sepsis in the 24 hrs prior to survey data	525		491		
Sepsis, Yes %(N)		30.5(160)		35.8(176)	0.069
ARDS in the 24 hrs prior to survey data	525		491		
ARDS, Yes %(N)		6.5(34)		7.7(38)	0.433
Severity of illness score in 24 hrs prior to survey date**	521		396		
APACHE II [median(IQR)]**		15.0(10,21)	269	18.0(12,24)	0.069
SAPS II [median(IQR)]**	N/A		88	42.0(28,52)	
SAPS III [median(IQR)]**	N/A		9	52.0(47,57)	
Severity of illness score, OTHER type **	N/A		30	6.1(30)	
Severity of illness, High(>= median)**		48.6(253)	396	56.3(223)	0.295
APACHE II chronic health points (CHP) criteria, %(N)	523		490		
CHP liver criteria		5.5(29)	486	6.0(29)	0.774
CHP renal criteria		4.0(21)	487	2.5(12)	0.166
CHP cardiac criteria		8.4(44)	487	8.4(41)	0.997
CHP respiratory criteria		7.1(37)	488	6.1(30)	0.554
CHP immunocompromised		8.6(45)	487	6.8(33)	0.277

Summary statistics of continuous variables are presented as median (IQR) with p values of non-parametric test (i.e. Wilcoxon rank-sum test). Summary statistics of categorical variables are presented in proportions with p values of chi^2 test. **Variables have >10% missing values. N/A= not applicable as data wasn't collected.
