# Supplementary material for: Patterns of intravenous fluid resuscitation use in adult intensive care patients between 2007 and 2014: An international cross-sectional study
Source: PLoS One. 2017 May 12;12(5):e0176292. doi: 10.1371/journal.pone.0176292 (PMC5428917; doi:10.1371/journal.pone.0176292)
Supplement: S8 Table — (PDF) [file pone.0176292.s009.pdf]

S8 Table. Comparison of indication for fluid and fluid prescriber between 1167 fluid resuscitation episodes in 2007 and 960 fluid resuscitation episodes in 2014

Variable	2007		2014		
	N	Summary statistics	N	Summary statistics	p value
**Indication for fluid in each fluid resuscitation, % (N)**	1152		955		
**Impaired perfusion/low cardiac output**		41.7 (480)		55.1 (526)	<0.001
**Ongoing bleeding**		5.7 (66)		2.9 (28)	
**Other fluid losses**		2.8 (32)		6.9 (66)	
**Unit protocol**		11.3 (130)		8.5 (81)	
**Abnormal vital signs**		35.6 (410)		23.8 (227)	
**Other**		3.0 (34)		2.8 (27)	
**Fluid prescriber, % (N)**	1167		960		
**Registrar (mid-level)**		35.6 (415)		34.9 (335)	<0.001
**Specialist (Consultant/Attending)**		24.9 (291)		34.5 (331)	
**Resident (Junior)**		34.9 (407)		26.1 (251)	
**Nurse**		2.8 (33)		3 (29)	
**Other**		1.8 (21)		1.5 (14)	
