# Supplementary material for: Patterns of intravenous fluid resuscitation use in adult intensive care patients between 2007 and 2014: An international cross-sectional study
Source: PLoS One. 2017 May 12;12(5):e0176292. doi: 10.1371/journal.pone.0176292 (PMC5428917; doi:10.1371/journal.pone.0176292)
Supplement: S9 Table — (PDF) [file pone.0176292.s010.pdf]

S9 Table. Sensitivity analysis – multivariate analysis of factors associated with the use of crystalloid or colloid for the first fluid resuscitation episode in 2014

Characteristic	OR (95% CI) for receiving crystalloid	p_value	OR (95% CI) for receiving colloid	p_value
**Study region**		<0.001		<0.001
**France**	1.00		1.00	
**Australia**	0.21 (0.06 to 0.73)		2.44 (0.76 to 7.86)	
**Brazil**	9.93 (3.22 to 30.63)		0.16 (0.06 to 0.44)	
**Canada**	0.59 (0.17 to 2.02)		1.06 (0.34 to 3.28)	
**China**	0.13 (0.04 to 0.41)		9.91 (3.49 to 28.12)	
**Denmark**	0.72 (0.17 to 3.02)		0.70 (0.16 to 2.99)	
**Germany**	5.25 (1.42 to 19.33)		0.14 (0.04 to 0.50)	
**New Zealand**	0.36 (0.08 to 1.56)		1.80 (0.44 to 7.33)	
**Saudi Arabia**	1.37 (0.22 to 8.55)		0.69 (0.11 to 4.31)	
**UK**	2.83 (0.37 to 21.72)		0.19 (0.03 to 1.14)	
**Other European countries**	0.24 (0.06 to 0.97)		1.91 (0.51 to 7.06)	
**Other countries**	0.59 (0.14 to 2.41)		1.67 (0.51 to 5.47)	
**Age (per one year increase)**	0.99 (0.98 to 1)	0.077	1.01 (0.99 to 1.02)	0.234
**Number of days in ICU at survey date**				
**Number of days in ICU at survey date =0 day**	1.00		1.00	
**Number of days in ICU at survey date >0 day**	0.4 (0.24 to 0.66)	<0.001	2.07 (1.28 to 3.33)	0.003
**Severity of illness in 24 hrs prior to survey date**				
**Low(< median)**	1.00	0.053	1.00	0.032
**High(>= median)**	0.72 (0.43 to 1.19)		1.24 (0.73 to 2.11)	
**Missing**	1.77 (0.81 to 3.87)		0.45 (0.21 to 1.01)	
**Trauma at hospital admission**				
**No Trauma**	1.00	0.116	1.00	0.050
**Trauma without TBI**	1.14 (0.53 to 2.44)		0.73 (0.34 to 1.58)	
**Trauma with TBI**	2.59 (1.04 to 6.41)		0.21 (0.06 to 0.73)	
**Sepsis in 24 hrs prior to survey date**				
**No**	1.00		1.00	
**Yes**	0.94 (0.62 to 1.41)	0.748	0.98 (0.67 to 1.42)	0.898
**Chronic health point liver criteria**				
**No**	1.00		1.00	
**Yes**	0.49 (0.21 to 1.16)	0.106	1.62 (0.79 to 3.33)	0.186
**Admission source**				
**Operating room after elective surgery**	1.00	0.007	1.00	<0.001
**Emergency room**	2.28 (1.12 to 4.61)		0.41 (0.21 to 0.78)	
**Hospital floor**	1.31 (0.66 to 2.6)		0.5 (0.26 to 0.97)	
**Transferred from other ICU or hospital**	1.36 (0.65 to 2.84)		0.72 (0.35 to 1.49)	
**Operating room after emergency surgery**	1.14 (0.6 to 2.19)		0.74 (0.4 to 1.37)	
**Hospital floor after previous ICU stay**	0.58 (0.28 to 1.23)		1.59 (0.8 to 3.18)	

**Indication for fluid**				
**Impaired perfusion or low cardiac output**	1.00	0.068	1.00	0.750
**Ongoing bleeding**	0.51 (0.14 to 1.83)		1.48 (0.51 to 4.28)	
**Other fluid losses**	0.97 (0.3 to 3.17)		1.10 (0.47 to 2.59)	
**Unit protocol**	0.48 (0.21 to 1.07)		1.63 (0.61 to 4.4)	
**Abnormal vital signs**	0.65 (0.43 to 0.99)		1.15 (0.8 to 1.64)	
**Indication for fluid, other**	0.21 (0.04 to 1.16)		2.37 (0.52 to 10.86)	
**Respiratory dysfunction**				
**No (SOFA<3)**	1.00		1.00	
**Yes (SOFA>=3)**	1.31 (0.8 to 2.14)	0.286	1.01 (0.66 to 1.55)	0.953
**Renal replacement therapy**				
**No**	1.00		1.00	
**Yes**	0.41 (0.22 to 0.79)	0.007	2.41 (1.13 to 5.12)	0.023
**Mechanical ventilation**				
**No**	1.00		1.00	
**Yes**	0.74 (0.46 to 1.2)	0.222	1.38 (0.87 to 2.19)	0.169
**Low filling pressure**				
**No**	1.00	0.232	1.00	0.464
**Yes**	1.52 (0.4 to 5.77)		1.33 (0.47 to 3.74)	
**missing**	1.45 (0.93 to 2.26)		0.81 (0.53 to 1.22)	
**Bilirubin**				
**<20 umol/L**	1.00	0.513	1.00	0.503
**>=20 umol/L**	0.75 (0.46 to 1.22)		1.32 (0.82 to 2.11)	
**missing**	0.85 (0.4 to 1.77)		1.06 (0.54 to 2.09)	
**Albumin**				
**<27 g/L**	1.00	0.717	1.00	0.181
**>=27 g/L**	0.88 (0.55 to 1.4)		0.88 (0.6 to 1.3)	
**missing**	1.14 (0.58 to 2.22)		0.57 (0.31 to 1.03)	
**Fluid output**				
**<1 ml/kg/hr**	1.00	0.153	1.00	0.024
**>=1 ml/kg/hr**	0.6 (0.35 to 1.03)		1.83 (1.09 to 3.07)	
**missing**	0.79 (0.38 to 1.64)		1.16 (0.59 to 2.29)	

Results are generated from a generalised estimating equation model with site/ICU ID as a cluster. When the P-values of the individual variable odds ratios (not presented) were <0.01 the odds ratios displayed in table are highlighted. The P-values displayed in the table are type III P-values. Analysis include 1,174 first fluid episodes from 1,174 study participants as data were lost due to missing values which could not be included in the multivariate analysis. This number represents a loss of 19.4% of first fluid episodes/study participants.
