# Supplementary figures and images for: Patterns of intravenous fluid resuscitation use in adult intensive care patients between 2007 and 2014: An international cross-sectional study
Source: PLoS One. 2017 May 12;12(5):e0176292. doi: 10.1371/journal.pone.0176292 (PMC5428917; doi:10.1371/journal.pone.0176292)

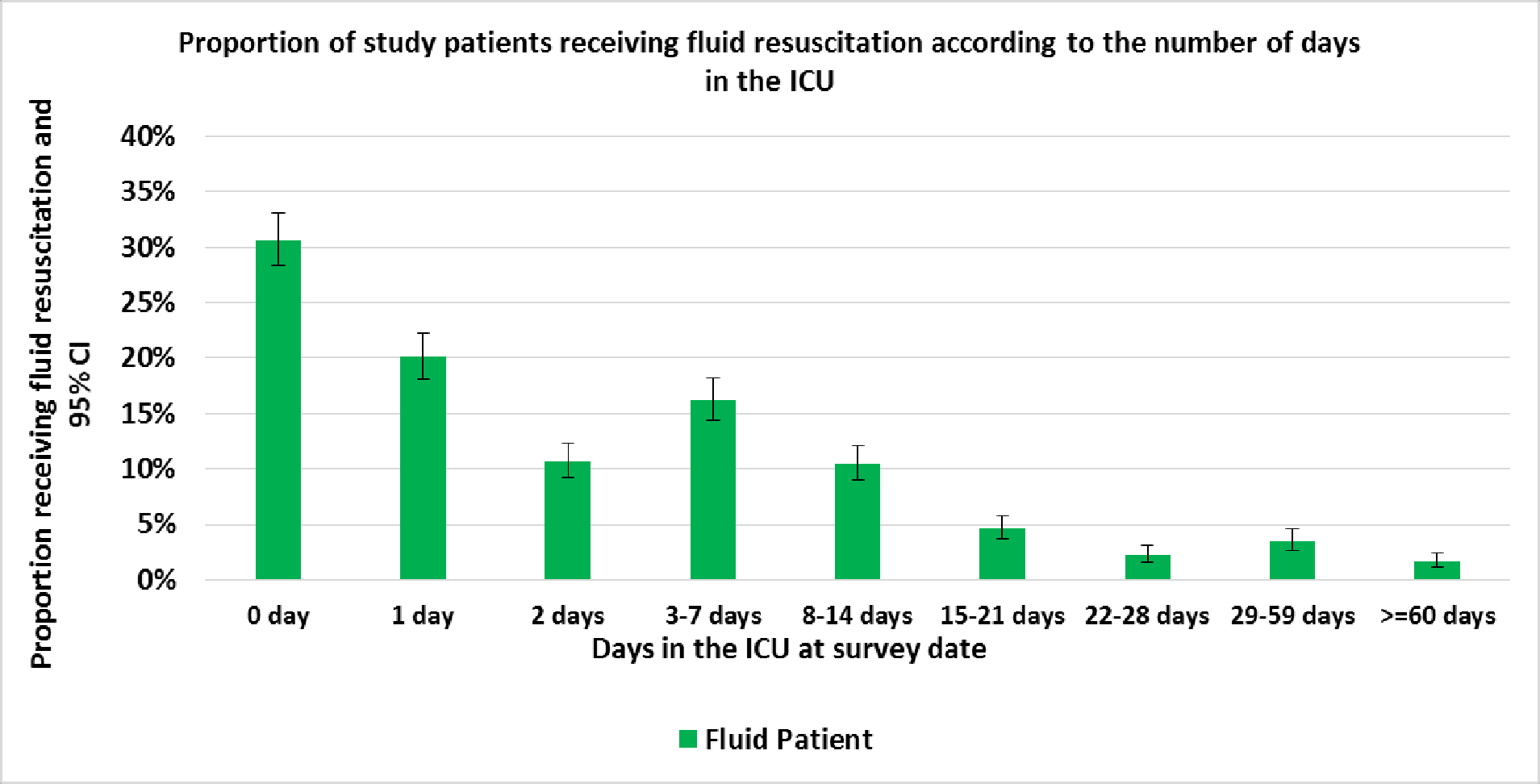

Supplement: S1 Fig — (TIF) [file pone.0176292.s011.tif]

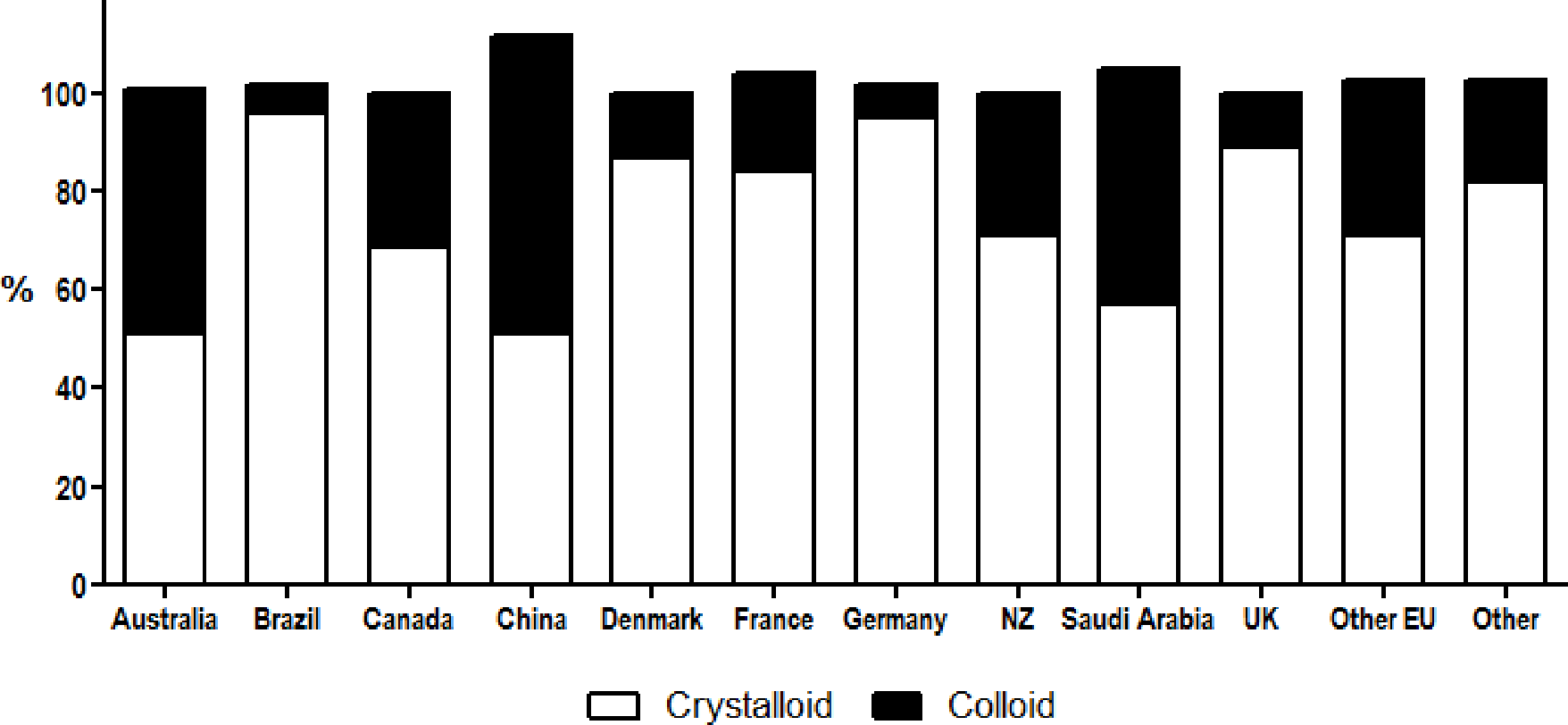

Supplement: S2 Fig — (TIF) [file pone.0176292.s012.tif]

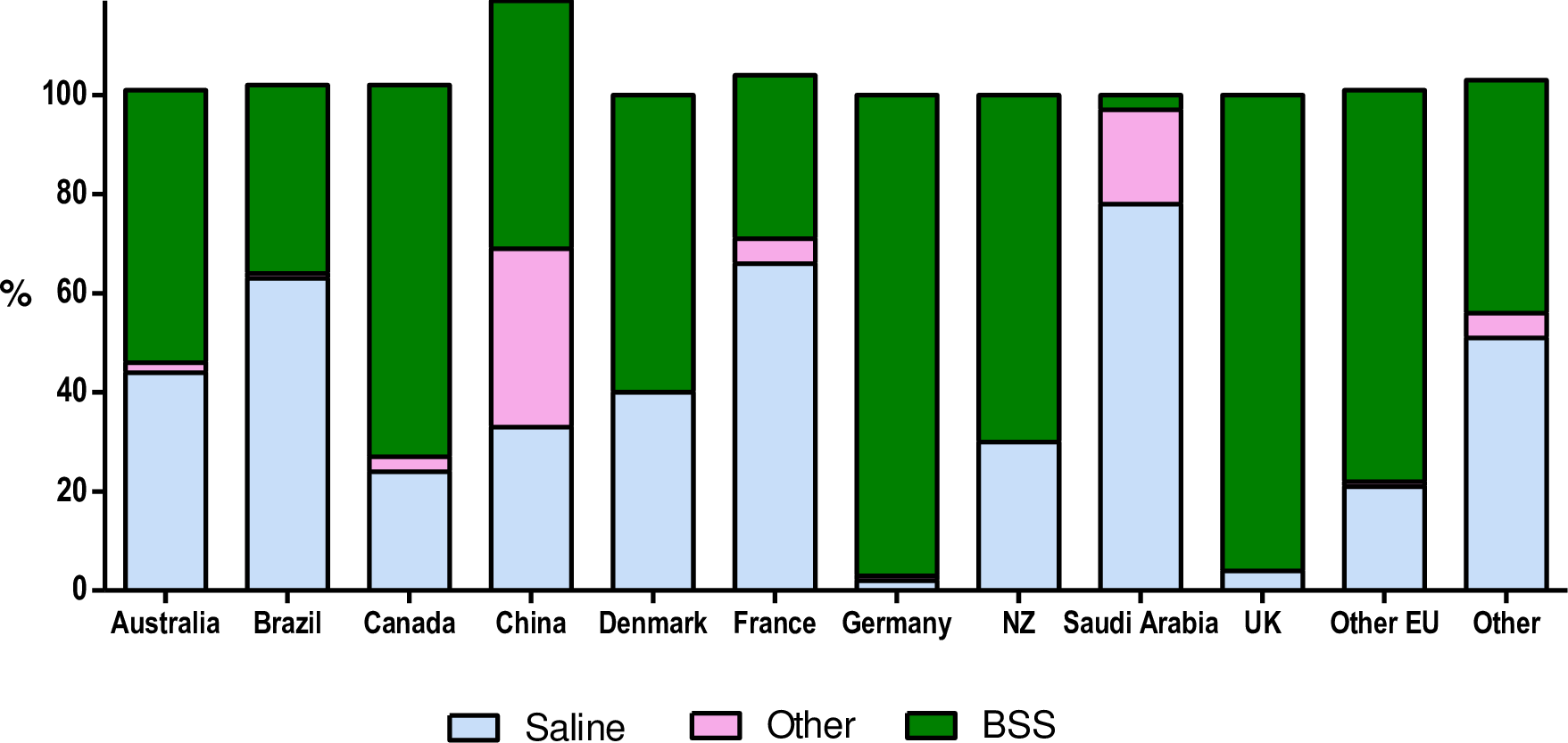

Supplement: S3 Fig — (TIF) [file pone.0176292.s013.tif]

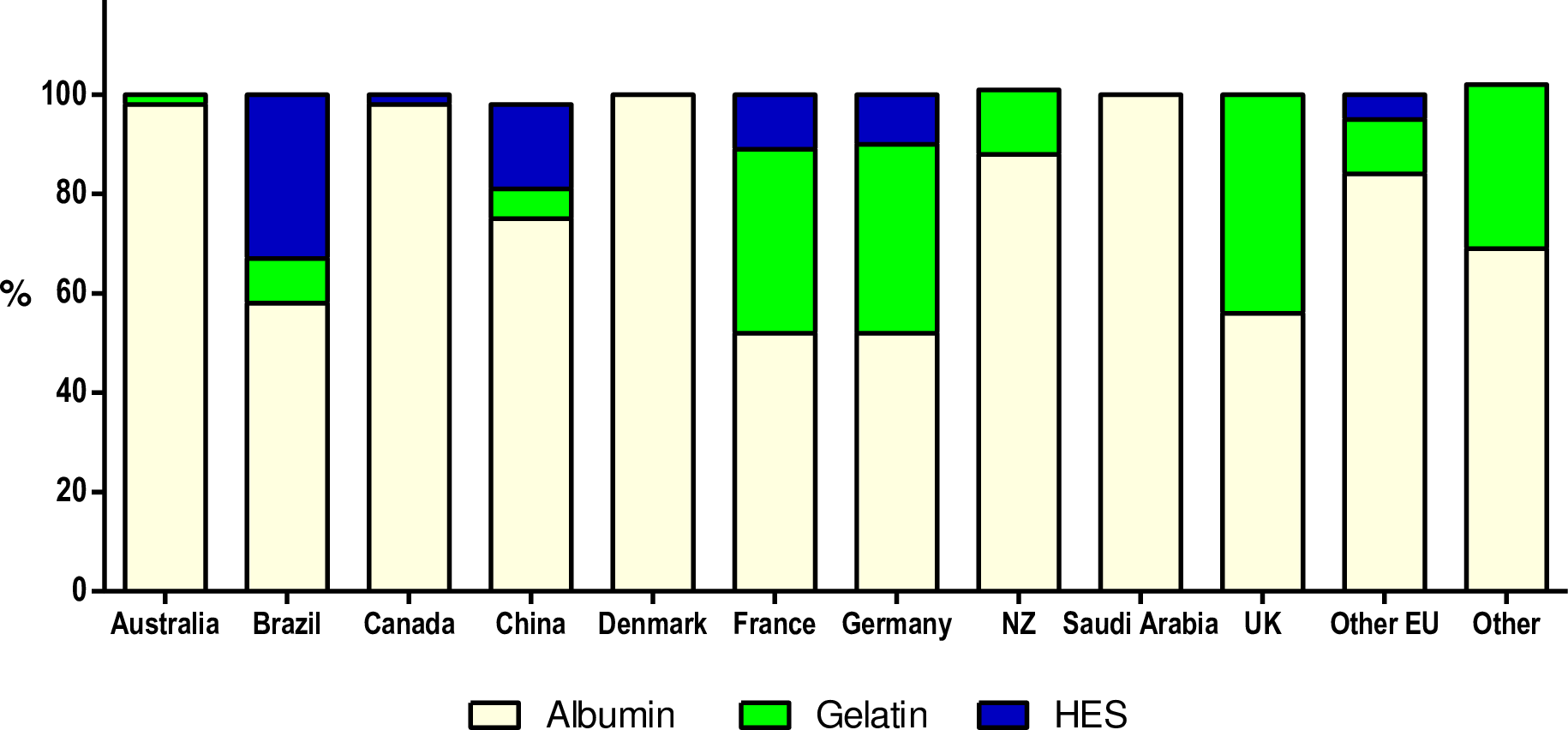

Supplement: S4 Fig — (TIF) [file pone.0176292.s014.tif]

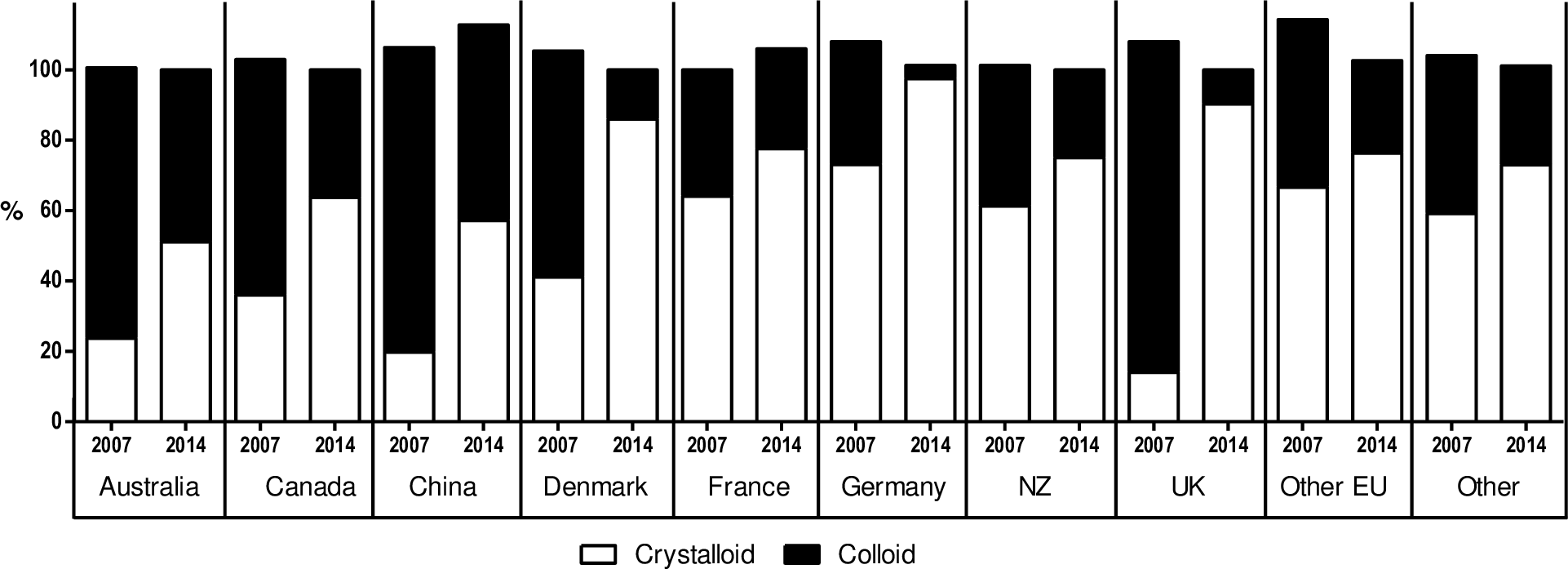

Supplement: S5 Fig — (TIF) [file pone.0176292.s015.tif]

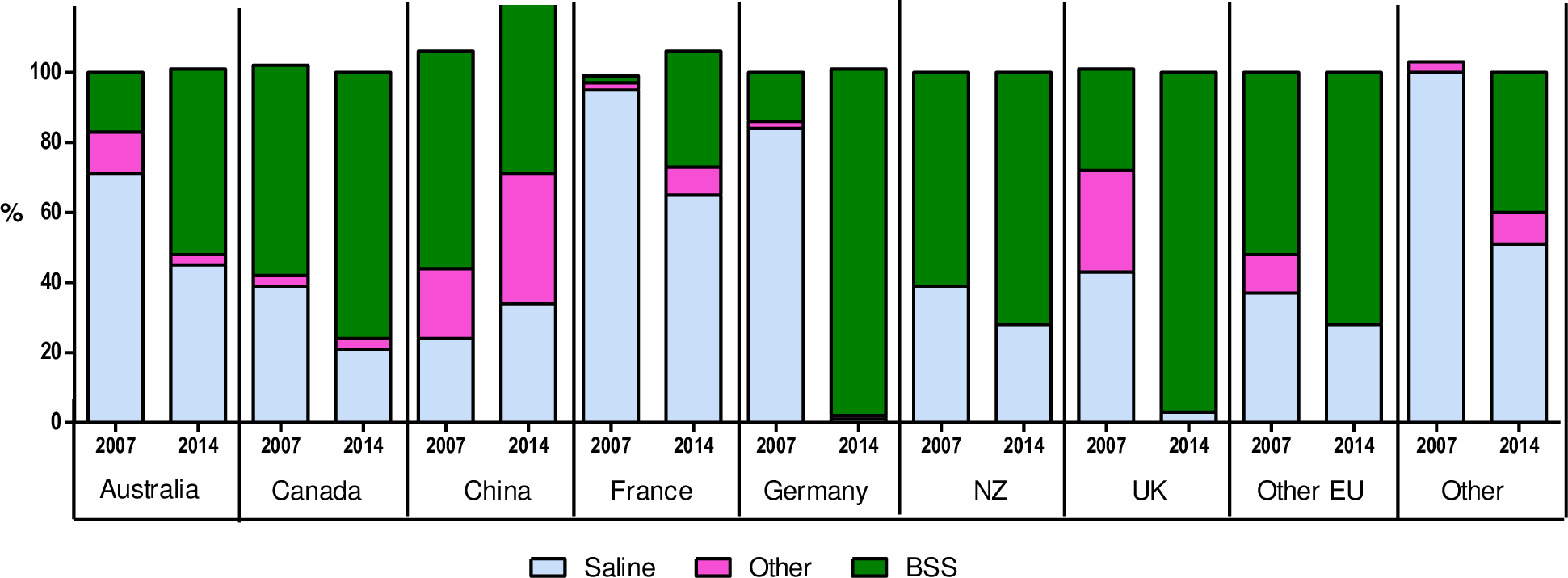

Supplement: S6 Fig — (TIF) [file pone.0176292.s016.tif]

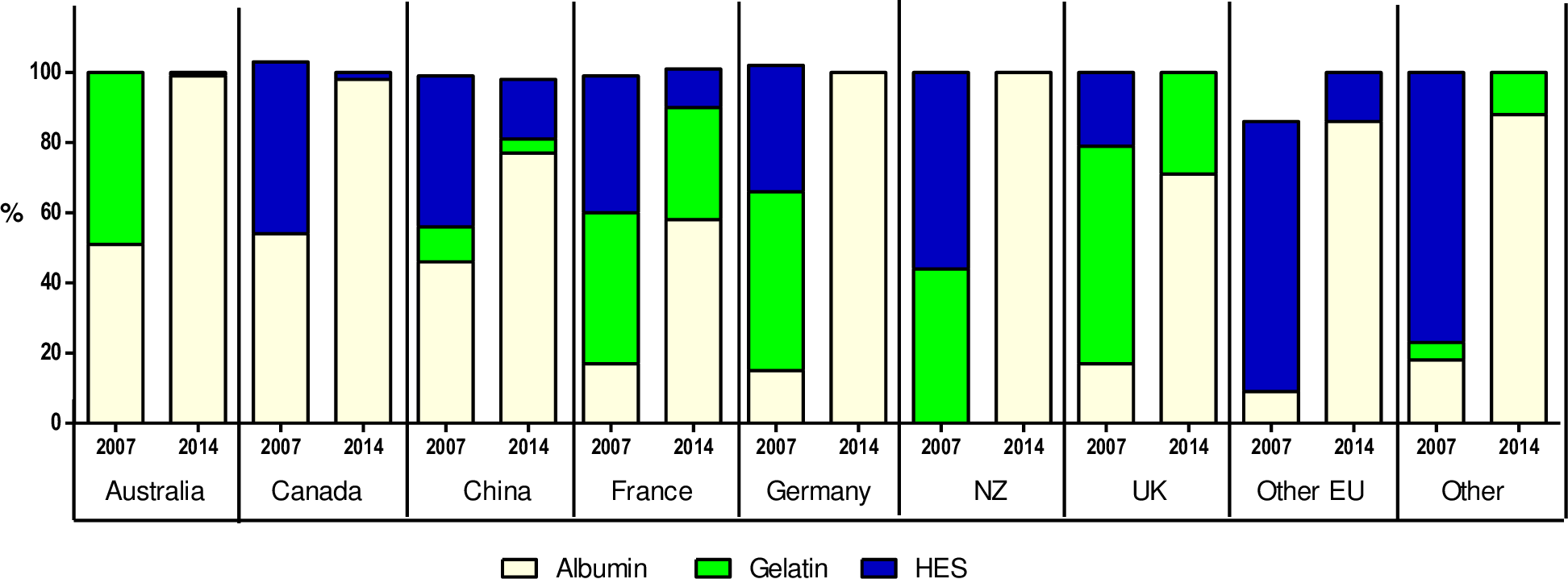

Supplement: S7 Fig — (TIF) [file pone.0176292.s017.tif]

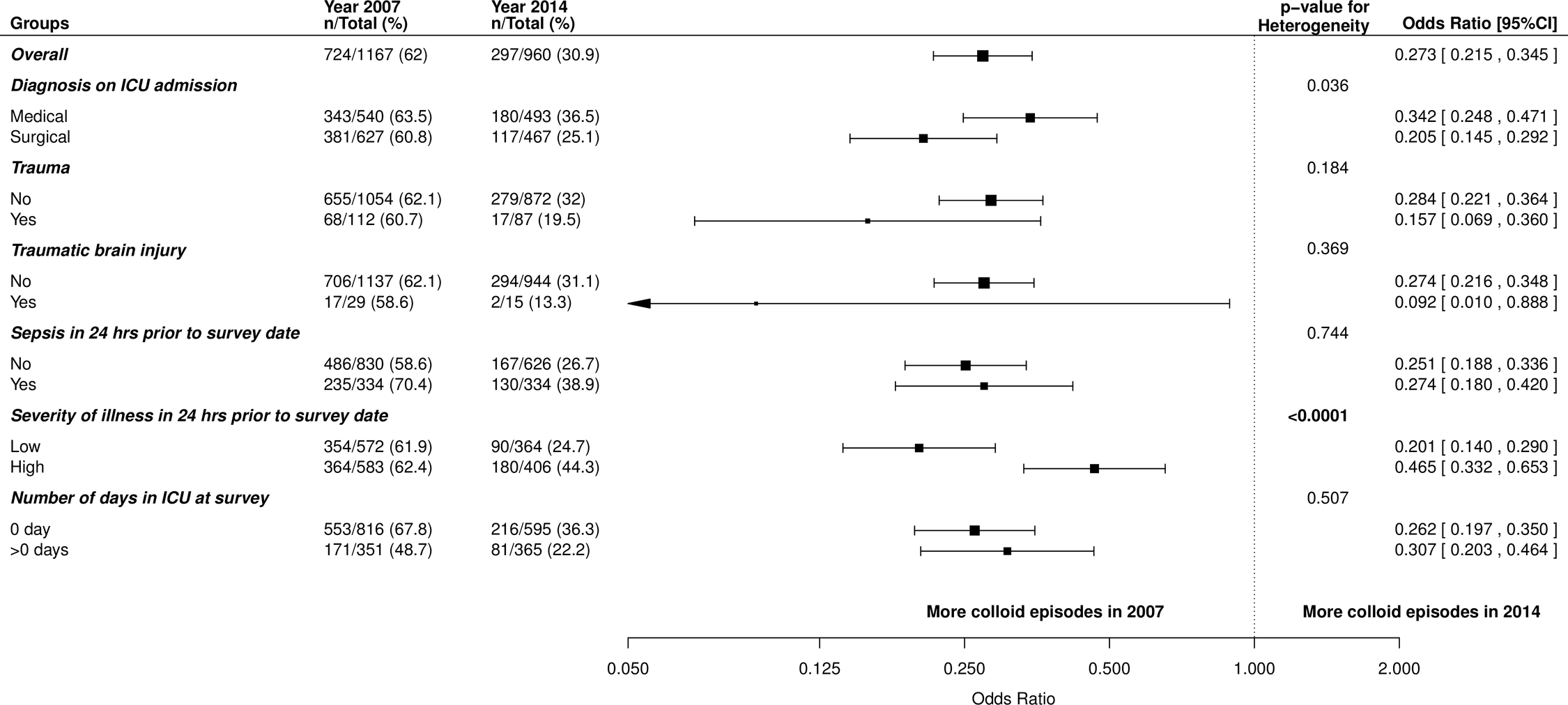

Supplement: S8 Fig — (TIF) [file pone.0176292.s018.tif]

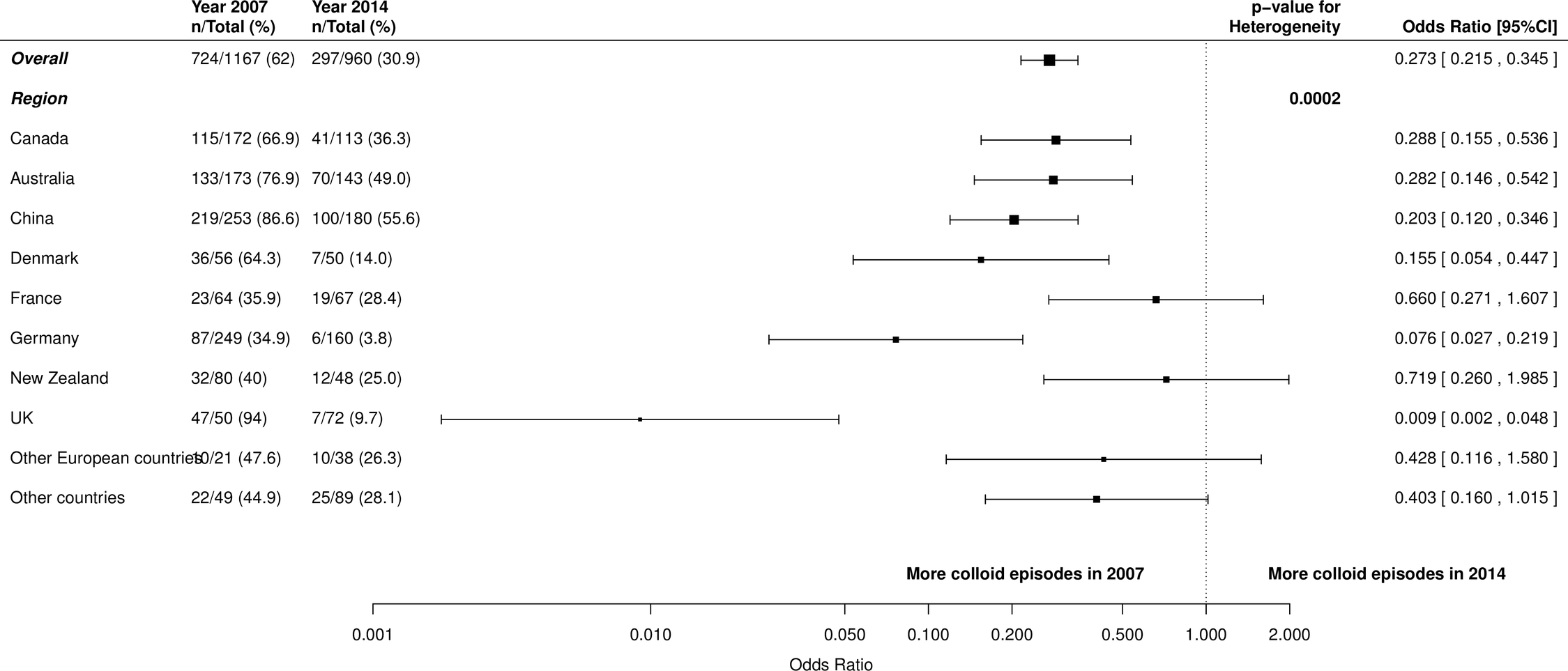

Supplement: S9 Fig — (TIF) [file pone.0176292.s019.tif]
